# Sensitive and Spectral Interference-Free Determination of Rhodium by Photochemical Vapor Generation Inductively Coupled Plasma Mass Spectrometry

**DOI:** 10.1021/acs.analchem.4c05921

**Published:** 2025-02-10

**Authors:** Karolína Hašlová, Stanislav Musil

**Affiliations:** † Institute of Analytical Chemistry of the Czech Academy of Sciences, Veveří 97, 602 00 Brno, Czech Republic; ‡ Charles University, Faculty of Science, Department of Analytical Chemistry, Hlavova 8, 128 43 Prague, Czech Republic

## Abstract

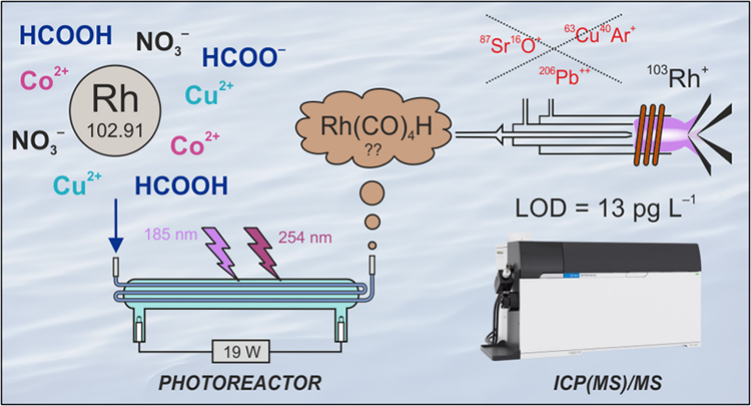

A sensitive method for Rh determination was developed by coupling photochemical vapor generation (PVG) for sample introduction to inductively coupled plasma mass spectrometry (ICPMS). PVG was conducted in a thin-film flow-through photoreactor operated in a flow injection mode from a photochemical medium comprising 10 M HCOOH. PVG efficiency was substantially enhanced by the addition of 10 mg L^–1^ Cu^2+^ and 5 mg L^–1^ Co^2+^ as mediators as well as 50 mM NaNO_3_. The volatile product (likely Rh(CO)_4_H) was found to be less stable when in prolonged contact with the liquid medium at the output from the photoreactor. Hence, further enhancement was achieved by introducing an Ar carrier gas near the exit of the photoreactor to minimize the interaction of volatile species with the liquid medium. Despite PVG efficiency reaching only 15%, measurement at the ultratrace level (20 ng L^–1^) was characterized by very good repeatability of peak area response (2.9%) and outstanding limits of detection (13 pg L^–1^, 6.5 fg absolute) using He in the collision cell. Interferences from potential coexisting metals, inorganic acids, and their anions were investigated. Accuracy was verified by analysis of OREAS 684 (Platinum Group Element Ore) and SRM 2556 (Used Auto Catalyst) following peroxide fusion for sample preparation. Application to the direct analysis of real river and lake water samples and reference materials AQUA-1 and SLRS-6 demonstrated excellent selectivity of the PVG-ICPMS methodology over conventional pneumatic nebulization-ICP(MS)/MS, the results of which were seriously biased by polyatomic interferences, especially from Sr and Cu, despite the use of various reaction/collision cell modes.

## Introduction

Rhodium is a noble metal and one of the rarest elements in the Earth’s crust.^1^ It belongs to the platinum group elements (PGEs) and also to a newly established group of so-called technology-critical elements (TCEs) defined by several common features, such as being rare in nature but increasingly used in modern technologies.^2^ With its corrosion-resistance, high reflectivity, and catalytic properties, Rh is utilized in a variety of applications, primarily in catalytic converters, but also in organometallic chemistry applications and the glass industry, wherein the demand for Rh is steadily rising.^1^ Its increasing usage has raised some issues of its impact on biogeochemical and environmental cycles as well as potential human health concerns.^3,4^ Due to environmental and socioeconomic aspects, the recycling and recovery of Rh have also attracted considerable interest in recent years.^5,6^

Inductively coupled plasma mass spectrometry (ICPMS) with pneumatic nebulization (PN) of liquid solutions has been preferentially employed for Rh determinations due to the speed of analysis and very high sensitivity.^7,8^ However, Rh is monoisotopic (^103^Rh), and although there are no atomic isobaric overlaps, the accuracy of determination can be compromised by polyatomic (CuAr^+^, SrO^+^, RbO^+^, ZnAr^+^, and others) and doubly charged ion (Pb^++^) spectral interferences contributing to ^103^Rh^+^ response. The influence of these interferences becomes especially serious at ultratrace Rh concentrations, typical of environmental samples, when the relative contribution from the interferent to apparent analyte signal intensity is large. For quadrupole-based instruments (single or triple), the use of intervening collision (typically He) or reaction (O_2_ or NH_3_) gases in a reaction/collision cell^9^ may help to overcome many of them, while the use of high-resolution ICPMS, available with a sector field analyzer, appears to not always achieve sufficient resolving power to separate ^103^Rh^+^ from the aforementioned molecular ions, especially ^87^Sr^16^O^+^.^8^

A substantial improvement in sensitivity and selectivity can be realized when ICPMS is coupled to vapor generation (VG) as a sample introduction technique.^10^ Analyte in the liquid medium is converted to a volatile compound (species), which is followed by its release from the liquid matrix and efficient gas phase transport to the detector. Analyte introduction efficiency can ideally be 100%, significantly better than that typically achieved with PN (<10%). Equally noteworthy is that the analyte is selectively separated from the sample matrix components, thereby eliminating spectral and nonspectral interferences during detection.

Among VG techniques, photochemical vapor generation (PVG) has gained significant importance and is still expanding regarding the number of analytes (elements) to which it can be applied.^11^ This group currently includes not only Hg and elements typically forming volatile hydrides but also nonmetals and many transition metals. Volatile analyte species are synthesized during ultraviolet (UV) irradiation of an aqueous photochemical medium containing low molar mass carboxylic acids, particularly formic and acetic acids, typically by using a low-pressure Hg lamp. Their photolysis yields strongly reducing aquated electrons (e_(aq)_^–^) and radical species (H^•^, R^•^, and CO_2_^•–^) that subsequently interact with the ionic analytes to form volatile free atoms (Hg^0^), hydrides, alkylated derivatives, or carbonyls, depending on the element and photochemical medium used.^12^ An important impetus for the further development of PVG and its extension to new analytes was the introduction of transition-metal ion mediators (sometimes called sensitizers), namely Cd^2+^, Co^2+^, Cu^2+^, Fe^2+/3+^, Mn^2+^, Ni^2+^ and V^4+/5+^,^13−28^ as well as the increasing use of advanced thin-film flow-through photoreactors^29^ that allow efficient irradiation of the sample with access to intense 185 nm radiation in addition to 254 nm. Conversely, the deleterious presence of concomitant inorganic acids and their anions, such as NO_3_^–^ and Cl^–^, in real samples is recognized as a major shortcoming of PVG techniques and is attributed to the quenching of free radicals during generation.^11,12^ Some exceptions are PVG of Se,^30−32^ Ge,^33^ and Sn,^34,35^ where significant positive effects of NO_3_^–^ or Cl^–^ on PVG efficiency were reported.

Recently, significant attention has also been paid to the application of PVG for the enhanced determination of PGEs. Osmium was the first element generated under oxidative PVG conditions,^36,37^ from pure water or in the presence of HNO_3_, as volatile OsO_4_.^38^ Following these studies, de Oliveira et al.^26^ proved that high yield of Os volatile species can also be obtained under photoreductive conditions using HCOOH or CH_3_COOH media containing Fe^2+/3+^ as the mediator. The first PVG of Rh (and possibly also Pd and Pt) from a mixture of HCOOH and CH_3_COOH was demonstrated in a pioneering work by Guo et al.,^39^ within a multielement interrogation of the feasibility of PVG. It was followed by a paper by de Oliveira and Borges,^25^ who investigated PVG of Ir, Pd, Pt, and Rh from an HCOOH medium using a photoreactor consisting of two low-pressure Hg lamps to irradiate a quartz tube through which the sample was passed. Focusing on seawater analysis, they described a positive effect of Cu^2+^ on the PVG efficiency of Ir and Rh. However, the limits of detection (LODs), obtained by coupling PVG with ICPMS, were not better than 20 ng L^–1^, most likely due to low PVG efficiencies. Extensive work was also undertaken in our laboratory on PVG of Ru^16^ and Ir^15^ from HCOOH media in the presence of Co^2+^ and Cd^2+^ mediators and using the thin-film flow-through photoreactor. High PVG efficiencies (near 30 and 90%, respectively) and correspondingly low LODs were achieved, even at single-digit pg L^–1^ levels. Such LODs are otherwise difficult to achieve with conventional approaches. Parallel to our PVG of Ru,^16^ Yang et al.^17^ described the simultaneous PVG of Ru and Os employing Co^2+^ and Cd^2+^ mediators as well. Very recently, PVG of Ru and Ir was found to be surprisingly efficient using very dilute HCOOH media (around 0.01 M),^40^ providing some additional insight into mechanistic aspects of PVG of these metals while leaving the potential for analytical application aside.

Obviously, PVG of Rh has not been the subject of sufficient research so far that could lead to a satisfactorily efficient PVG and provide a basis for its ultrasensitive determination. Therefore, the motivation for this study was to utilize the thin-film flow-through photoreactor and identify PVG conditions that would result in efficient and repeatable PVG yields. Ideally, the method should be able to determine Rh at natural or only slightly contaminated concentration levels and, equally important, with minimal influence of spectral interferences, which is a major limiting factor of ICPMS determination based on conventional PN.

## Experimental Section

### Instrumentation

The PVG system, as described recently,^15,16^ was based on a flow injection (FI) mode of operation and consisted of a chemifold with an injection valve (0.5 mL sample volume), a 15 mL plastic gas-liquid separator (GLS), and a thin-film flow-through photoreactor (19W low-pressure Hg discharge lamp, Jitian Instruments Co., China) that provides access to both 185 and 254 nm Hg emission lines for sample irradiation. Details of the entire setup are given in the Supporting Information, Figure S1. The important dimensions of the whole photoreactor and possible configurations for the introduction of an Ar carrier downstream of the photoreactor (standard or modified side outlet arm enabling fast stripping of the volatile species) are described in Supporting Information, Figure S2. A 300 mL min^–1^ flow of Ar carrier was found to efficiently release and transport volatile Rh species from the GLS to the ICPMS (details in the Supporting Information).

An Agilent 7700x single quadrupole ICPMS was typically employed during experiments focused on the optimization of PVG parameters, while an advanced Agilent 8900 triple quadrupole ICPMS was used for the evaluation of analytical figures of merit, PVG efficiency, and analytical applications. Measurements with FI-PVG were conducted in time-resolved analysis mode using He gas (4.1 mL min^–1^) in the reaction/collision cell. Optimal plasma settings for PVG measurements with the Agilent 8900 ICPMS and the selected isotopes are summarized in Table S1. Due to the very similar construction of a spray chamber, nebulizer, and conduit to the torch, the gas flow setting used with the Agilent 7700x ICPMS was the same. All results were based on the ^103^Rh peak area response. The measurement and evaluation procedure and established conventions are described in the Supporting Information.

### Reagents and Materials

Deionized water (DIW, <0.2 μS cm^–1^, Ultrapur, Watrex) was used for the preparation of all solutions. Formic acid (98%, p.a., Lach-Ner, Czech Republic) was used for the formulation of photochemical media of various molarities (M, i.e., mol L^–1^). Sodium nitrate (99.99%, Suprapur) from Merck was used as an additive to the photochemical medium. Unless otherwise stated, both DIW and HCOOH were sub-boil distilled. A commercial stock analytical standard solution of 1000 mg L^–1^ Rh^3+^ (as RhCl_3_) in 5% (w/w) HCl was obtained from Fluka. Specifications of additional analytical standards, compounds used as potential metal ion mediators, and other chemicals are given in the Supporting Information.

### Sample Preparation

A pulverized OREAS 684 Platinum Group Element Ore certified reference material (CRM) produced by Ore Research & Exploration Pty Ltd. (Australia) and Standard Reference Material (SRM) 2556 (Used Auto Catalyst, Pellets) from the National Institute of Standards and Technology (NIST) were decomposed by means of sodium peroxide fusion. Since SRM 2556 is hygroscopic and contains approximately 4.2% moisture content and some organic matter,^41^ this material was dried/calcined at 500 °C for 2 h, following directions in the certificate. Approximately 0.2 g of each material was mixed with 2 g of Na_2_O_2_ in a sintered alumina crucible (27 mL). Fusion was carried out in a muffle furnace, and the heating program was as follows: 5 min ramp to 200 °C, 15 min hold, 10 min ramp to 650 °C, and 40 min hold, after which it was cooled to ambient temperature. The resulting vitreous sample mass was carefully dissolved in 20 mL 5 M HNO_3_ and 30 mL DIW by alternately adding them in 5 mL increments. The digests of OREAS 684 and SRM 2556 were further diluted (200-fold and 4000-fold, respectively) and prepared in a photochemical medium comprising 10 M HCOOH, 50 mM NaNO_3_, with 10 mg L^–1^ Cu^2+^ and 5 mg L^–1^ Co^2+^ as mediators for analysis by FI-PVG-ICPMS. For comparison, the digests were also prepared in 2% (w/v) HNO_3_ and analyzed by conventional PN-ICP(MS)/MS.

Two water CRMs, AQUA-1 (drinking water) and SLRS-6 (river water), from the National Research Council Canada, were also analyzed by FI-PVG-ICPMS and, for comparison, by conventional PN-ICP(MS)/MS employing various modes of operation of the reaction/collision cell. These are detailed in the Supporting Information, Table S2. Additionally, a river water sample was collected from the Vltava river in Prague and alpine lake water was sampled from the Jägersee (Kleinarl, Austria). These samples were filtered through a 0.45 μm PTFE filter and prepared with no dilution except for the addition of concentrated HCOOH and solutions of mediators and NaNO_3_ for analysis by FI-PVG, or adjusted to 2% (w/v) HNO_3_ for measurements using PN sample introduction.

## Results and Discussion

### PVG without Mediators

HCOOH has been almost exclusively employed as the photochemical medium for PVG of transition metals, ensuring strongly reductive PVG conditions.^11,12^ The PVG of Rh was thus first examined using only a HCOOH-based medium, without transition metals as mediators or other additives, to establish a “baseline” performance. The effects of the most important parameters influencing PVG, i.e., concentration of HCOOH and sample irradiation time (IT), which with FI-PVG is inversely proportional to the sample flow rate through the photoreactor, are depicted in Figure S3A,B. Employing a set sample flow rate of 1.5 mL min^–1^ through the photoreactor, the peak area response for 40 μg L^–1^ Rh gradually increased with higher HCOOH concentrations and reached a maximum at 8–10 M HCOOH, beyond which it significantly declined (Figure S3A). No second maximum of the PVG efficiency was evident at low concentrations of HCOOH (around 0.01 M) as reported recently for Ru, Re, and Ir.^40^ Using 10 M HCOOH, sample flow rates were then varied in the range 0.5–2.5 mL min^–1^, yielding maximum peak area response at flow rates 1.25–1.5 mL min^–1^ (Figure S3B), corresponding to ITs of 29–35 s. Although at higher sample flow rates, i.e., shorter IT, the lower responses suggest that the irradiation of the sample is insufficient, the decline at longer ITs can be explained only by poor photostability of volatile species of Rh or their poor chemical stability when in contact with the liquid medium in the photoreactor outlet that is not continuously irradiated (see below).

Acetic acid-based media were found not effective for PVG of Rh, which is consistent with earlier observations on PVG of other transition metals, with the exception of Os^26^ and Re.^14^ Responses generated from 4 and 10 M CH_3_COOH at 1.5 mL min^–1^ were more than 350-fold lower than those obtained with 10 M HCOOH. The addition of 0.5 and 1 M CH_3_COOH to 9.5 and 9 M HCOOH, respectively, had no effect in comparison to 10 M HCOOH while the addition of 2 M CH_3_COOH to 8 M HCOOH caused a serious 70% decrease in response.

The preliminary experiments thus confirmed feasibility of PVG of Rh using only HCOOH; however, the overall PVG efficiency estimated when using a 10 M HCOOH medium and sample flow rate of 1.5 mL min^–1^, which provided the maximum peak area sensitivity, was <0.1%. Attempts to optimize pH by addition of liquid ammonia to partially neutralize solutions of 10 M HCOOH in an effort to enhance performance were unsuccessful (see Supporting Information, Figure S4).

### PVG in the Presence of Metal Ion Mediators

**Figure 1:**
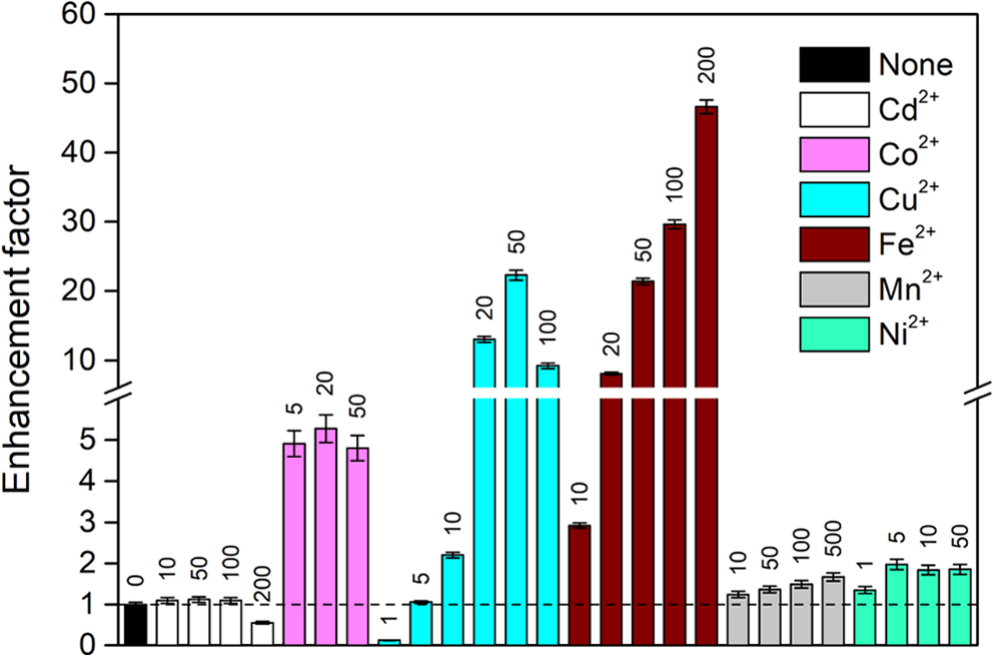
Enhancement effects from various metal ion concentrations for PVG from 2 μg L^–1^ Rh^3+^ in 10 M HCOOH at a sample flow rate of 1.5 mL min^–1^. The numbers above each column express the concentrations of metal in mg L^–1^, the dashed line corresponds to the enhancement factor of 1 (no mediator). Uncertainties are expressed as combined SD (*n* ≥ 3).

The possibility of substantial enhancement in overall PVG efficiency was examined by the addition of various metal ion mediators (Cd^2+^, Co^2+^, Cu^2+^, Fe^2+^, Mn^2+^, and Ni^2+^), based on their previously reported efficacy for PVG of especially transition metals as analytes.^13−27^ The metals were added only to the Rh^3+^ standard in 10 M HCOOH and not to the carrier (10 M HCOOH) into which the standard was injected. The PVG enhancement factors attained with various concentrations of individual metal ions using a sample flow rate of 1.5 mL min^–1^ are shown in Figure 1.

Significant enhancement effects were exhibited by Co^2+^, Cu^2+^, and Fe^2+^ while other ions had markedly lower effects (≤2). There was no clear maximum identified for the addition of Co^2+^ as the mediator and the enhancement factors amounted to 4.9–5.3-fold in the range of 5–50 mg L^–1^. A quite extraordinary behavior was evident at 1 mg L^–1^ Cu^2+^ which suppressed the PVG of Rh by around 8-fold. However, at >5 mg L^–1^ Cu^2+^ the PVG efficiency was significantly enhanced and a clear maximum was attained at 50 mg L^–1^ resulting in an enhancement factor of ≈22. A similar impact of added Cu^2+^ was observed in the pioneering work by de Oliveira and Borges,^25^ who reported the greatest enhancement from 10 mg L^–1^ but they used significantly different conditions for sample irradiation available with their photoreactor (quartz capillary attached to 40W low-pressure Hg lamps utilizing mainly 254 nm emission line for irradiation). The greatest enhancement arose with Fe^2+^, wherein the peak area response of Rh gradually increased with Fe^2+^ concentration up to 200 mg L^–1^ Fe^2+^ to provide an enhancement factor of ≈47. Despite this great effect, higher concentrations were not tested, and Fe was not considered for further analytical use because of several problems. Volatile Fe(CO)_5_ is generated^42,43^ concurrently with the volatile species of Rh but the PVG efficiency was estimated to be low (≈0.3%) under these PVG conditions. However, due to the very high concentrations of Fe^2+^ required to achieve this effect, this mediator still presents some loading to the plasma/interface. In fact, brownish deposits were typically visible at the tip of the torch injector after a full day of PVG measurements with ≥100 mg L^–1^ Fe^2+^. More importantly, significantly higher and quite persistent Rh PVG blanks were obtained after the PVG from 2 μg L^–1^ Rh standards containing Fe^2+^ as the mediator compared to, for example, using Cu^2+^. The blank corresponded to 50 ng L^–1^ Rh for the addition of 100 mg L^–1^ Fe^2+^ and very slowly declined. This high blank cannot be attributed to Rh contamination from the Fe^2+^ mediator because no Rh was detected in the stock solution of Fe^2+^ by conventional PN-ICPMS and the blank containing 100 mg L^–1^ Fe^2+^ measured in the “clean” system (cleaning procedure described in the Supporting Information) was low and comparable to that measured with 50 mg L^–1^ Cu^2+^. Since volatile species of Fe is not efficiently generated and the required Fe concentrations for PVG of Rh are high, it is likely that a significant amount of reduced Fe remains (semipermanently) deposited in the photoreactor. This is supported by visual observation of yellow-brownish “deposits” in the PTFE tubing and quartz photoreactor parts after several sample injections with 100 or 200 mg L^–1^ Fe^2+^. Thus, the reason for elevated blank values may arise from some kind of interaction between Fe and Rh, such as codeposition during PVG in the photoreactor.

**Figure 2:**
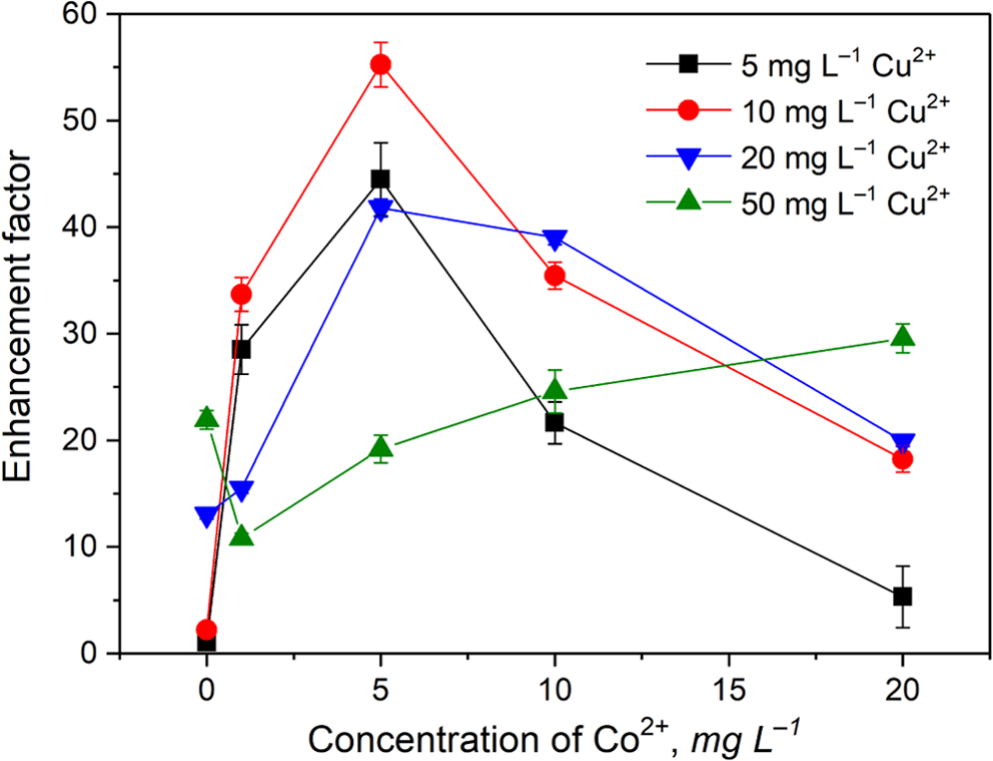
Effect of various combinations of Cu^2+^ and Co^2+^ ions present in 10 M HCOOH on PVG from 1 μg L^–1^ Rh^3+^ at a sample flow rate of 1.5 mL min^–1^. Uncertainties are expressed as combined SD (*n* ≥ 3).

An additional (synergistic) effect arising from the presence of two added metals to the photochemical medium has been demonstrated for PVG of several analytes recently, including Mo, Ir, Os, Re, Ru, As, and Te.^14−18,20,40,44^ In this work, only those metals that provided significant positive effects on the PVG of Rh (i.e., Co, Cu, and Fe) were assessed in combinations. A mixture of Cu^2+^ and Co^2+^ was identified as the most suitable due to the additional enhancement in PVG efficiency (Figure 2) and because no significant memory effects emerged and the blanks remained low.

It is evident that the maximum enhancement factor of ≈55 (relative to PVG response in the absence of both mediators) is reached at specific concentrations of Cu^2+^ and Co^2+^, namely 10 mg L^–1^ Cu^2+^ and 5 mg L^–1^ Co^2+^. Remarkably, the dependence on Co^2+^ concentration in the presence of 50 mg L^–1^ Cu^2+^ exhibits a clear minimum at 1 mg L^–1^ Co^2+^, illustrating the complex role of individual added mediators. Similar unexpected attenuation of the response was observed at 1 mg L^–1^ Cu^2+^ when used alone (Figure 1). For comparison, experiments were also focused on the effect of the copresence of Cu^2+^ and Fe^2+^. A combination of 20 mg L^–1^ Cu^2+^ and 200 mg L^–1^ Fe^2+^ resulted in an enhancement factor (≈56) very similar to that obtained with 10 mg L^–1^ Cu^2+^ and 5 mg L^–1^ Co^2+^, but it was not considered analytically useful because of the occurrence of the similar memory effects as described above for Fe^2+^ as the sole mediator.

The following optimization was thus based on the use of 10 mg L^–1^ Cu^2+^ and 5 mg L^–1^ Co^2+^ as mediators. Reoptimization of HCOOH concentration and sample flow rate with this combination did not reveal any shifts in optimal values, and clear maxima were still obtained at 8–10 M HCOOH and 1.25–1.5 mL min^–1^.

### Effect of the Residence Time of Volatile Species in the Photochemical Medium

It was demonstrated in our previous studies devoted to PVG of Mo,^21^ Ru,^16^ and Ir^15^ that the majority of the volatile species generated in our system (Figures S1 and S2A) is efficiently and rapidly released to the gas phase in the transfer PTFE tubing (1 mm i.d., 54 cm) after mixing with Ar carrier prior to reaching the bottom of the GLS. The high flow of the Ar carrier forms a thin film of liquid in this tube, which facilitates the release. This was also confirmed herein for Rh because the relative peak area response measured with the GLS that was operated in such a way that no liquid photochemical medium was maintained in the GLS (the tube for waste removal was moved to the bottom of the GLS) was 94 ± 5% compared to the standard setup of the GLS in which 1.5 mL of remaining photochemical medium was maintained.

Follow-up experiments fortuitously revealed the serious effect of the residence time of generated volatile species in the photochemical medium before their release into the gas phase by means of the introduction of the Ar carrier stream. The side quartz outlet arm (2 mm i.d., 5 cm) of the photoreactor accidentally broke off at the extremity of the photoreactor but was replaced by a PTFE tube of as short a length and volume as possible (0.5 mm i.d., 3 cm). This modification (cf. Figure S2A,B) led to a reduction in the volume of the conduit upstream of mixing with Ar carrier from an original ≈0.200 to ≈0.006 mL, corresponding to a decrease in time during which volatile species remained dissolved in the liquid medium from ≈8 s to ≈0.24 s (at the sample flow rate of 1.5 mL min^–1^). More importantly, the impact of this was an approximately 2-fold increase in the peak area response and thus the overall PVG efficiency.

In the following experiment, the effect of the residence time of the volatile species in the photochemical medium after exiting the photoreactor was examined in detail by varying the length (0, 10, 20, and 30 cm) and thus the volume of a PTFE tubing (1 mm i.d.) inserted between the new outlet and the T-piece for the introduction of the Ar carrier (Figure S5). The gradual decrease in response with added volume indicates that the generated volatile species is relatively unstable and decomposes after exiting the irradiated part of the photoreactor.

Due to this modification to the photoreactor, it was necessary to verify the effects of the basic PVG parameters (HCOOH concentration and sample flow rate). The experiments are detailed in the Supporting Information, Figure S6A,B and resulted in the final selected optimal values of 10 M HCOOH and 1.25 mL min^–1^.

### Effect of Nitrates

**Figure 3:**
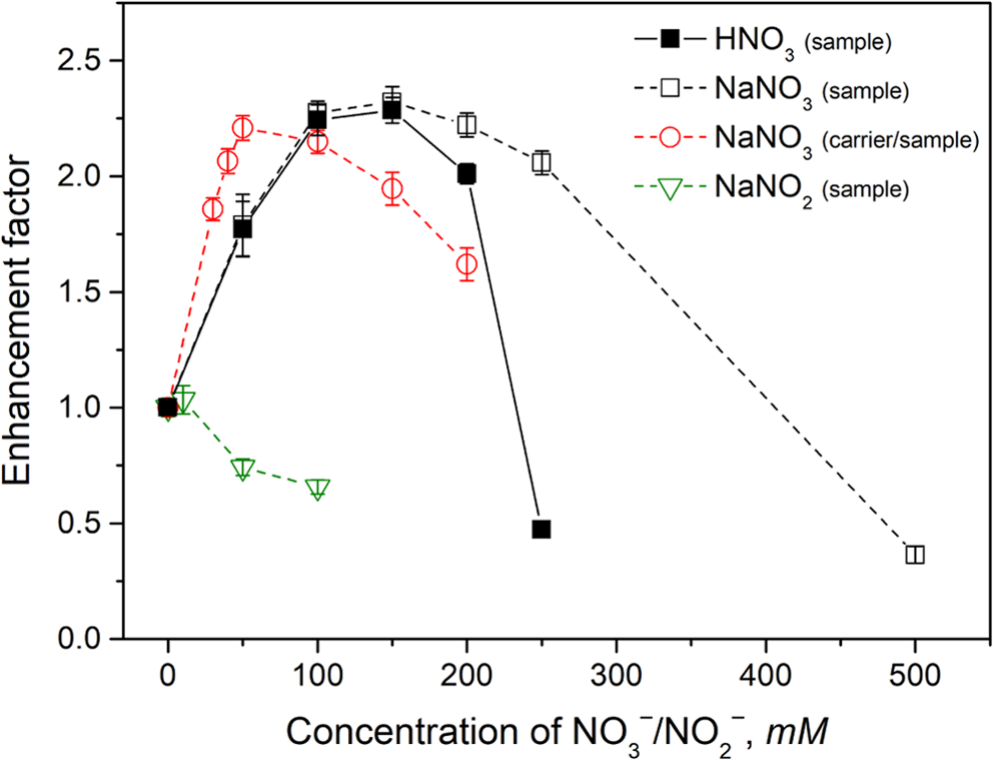
Effect of various concentrations of HNO_3_, NaNO_3_, and NaNO_2_ added to the sample (or both sample and carrier) prepared in 10 M HCOOH and containing 10 mg L^–1^ Cu^2+^ and 5 mg L^–1^ Co^2+^ as mediators on PVG from 0.25 μg L^–1^ Rh^3+^ at a sample flow rate of 1.25 mL min^–1^. Uncertainties are expressed as combined SD (*n* ≥ 3).

Nitrate anions usually present a serious interference with PVG methodology.^11,12^ Although a positive effect of the presence of NO_3_^–^ was reported for PVG of Se,^30−32^ the effects observed with our experimental setup have always been strongly negative for transition metals, even at concentrations as low as 1 mM,^21^ or 10 mM.^15,16,22,45^ However, in the present work, NO_3_^–^ was found to significantly enhance the PVG efficiency of Rh. As can be seen in Figure 3, the addition of 100–150 mM HNO_3_ to the Rh standard together with 10 mg L^–1^ Cu^2+^ and 5 mg L^–1^ Co^2+^ as mediators resulted in an enhancement of the peak area response by a factor of more than two. A similar maximum was achieved when NaNO_3_ was used instead of HNO_3_, hence, it is obvious that the increase in PVG efficiency is due to the presence of NO_3_^–^ and not to a change in the pH. The “pH effect” likely occurs at concentrations ≥150 mM as is indicated by the more serious decline of the response in HNO_3_ as compared to NaNO_3_. The similar positive effect of NO_3_^–^ was subsequently confirmed for PVG conducted in the absence of Cu^2+^ and Co^2+^ mediators. For comparison, no positive effect, but rather slightly negative, on PVG of Rh was observed with 10–100 mM NaNO_2_, which is an intermediate of NO_3_^–^ reduction during PVG.^11,46^ This means that the enhancement can be solely attributed to NO_3_^–^ and not to the species derived from the photolysis of NO_3_^–^ during PVG. The negative effects at 50 and 100 mM cannot be attributed only to the interference of NO_2_^–^ by quenching of free radicals and e_(aq)_^–^ but rather to degradation of the photochemical medium prior to PVG. Samples with such added NO_2_^–^ concentrations were visibly seen to become saturated with gases (likely NO and/or NO_2_) after NaNO_2_ addition, preventing delivery of a full volume of sample (0.5 mL) to the photoreactor due to many bubbles formed in the sample loop of the injection valve.

Following the previous observation of enhanced PVG due to the presence of NO_3_^–^ in a sample, the addition of NaNO_3_ to both the sample and the photochemical medium that serves as the carrier into which the sample is injected was examined. The dependence in Figure 3 indicates that the optimum NaNO_3_ concentrations shifted to 50–100 mM. This can, to some extent, be attributed to a dispersion of the injected analyte as well as NO_3_^–^ in the photoreactor conduit resulting in the NO_3_^–^ concentrations at the leading and falling edges of the analyte zone being lowered during their transport through the photoreactor. This is also supported by a relatively significant deterioration of measurement precision for 50 mM NO_3_^–^ added only to the sample; most probably, this concentration is close to the edge of the optimal range. A concentration of 50 mM NaNO_3_, added to both the sample and the photochemical medium (carrier), was thus selected as optimum.

Although the tentative explanation of the positive effect of NO_3_^–^ on PVG of Rh is not absolutely clear, some remarks on the mechanism of its action are discussed in the Supporting Information.

### Figures of Merit

The following PVG conditions were selected for routine use: 10 M HCOOH containing 50 mM NaNO_3_ as the photochemical medium delivered at 1.25 mL min^–1^ (IT = 35 s) and the addition of 10 mg L^–1^ Cu^2+^ and 5 mg L^–1^ Co^2+^ to the standard/sample. The PVG efficiency under these conditions was determined by a comparison of peak area sensitivities (slopes) obtained with both FI-PVG and FI-PN processed under the same plasma conditions.^15,16,18,22,40^ Considering a nebulization efficiency of 7.51 ± 0.05% that was determined using a dynamic mass flow approach^47^ under ICPMS conditions summarized in Table S1, the ratio of sensitivities reached 1.94 ± 0.01, resulting in overall PVG efficiency of 14.6 ± 0.1%. Attempts to further enhance the PVG efficiency by introducing air segments before and after the injected liquid sample zone, which was found useful in recent studies,^33,48,49^ resulted in only a small improvement (1.22-fold). This approach was not pursued further in an effort to keep the analytical procedure simple. Details are provided in the Supporting Information.

The repeatability (RSD) of the peak area response from a 20 ng L^–1^ Rh standard consecutively measured by FI-PVG-ICPMS methodology was very good (2.9%, *n* = 10). The calibration function for ^103^Rh using 0, 2, 5, 20, 100, and 250 ng L^–1^ Rh^3+^ standards was linear (*R*^2^ > 0.9999) resulting in an outstanding LOD (3σ, *n* = 11) of 13 pg L^–1^ (6.5 fg absolute). This LOD is 3 orders of magnitude lower than that reported by de Oliveira and Borges who focused on PVG of Rh for the analysis of seawater.^25^ For blank measurements, there was a typical small but persistent peak-shaped signal above the baseline, comprising single to a few tens of counts for 0.1 s dwell time, because the PVG efficiency is greatly enhanced in a zone of the injected sample/blank, where Cu^2+^ and Co^2+^ are present as the mediators. The LOD is thus driven by the variance of the peak area of this blank signal, the concentration of which corresponded to 0.05–0.10 ng L^–1^ (provided the PVG system is clean and not contaminated by prior measurement of a sample containing >20 ng L^–1^ Rh). Since sub-boil distilled HCOOH and DIW were used for the preparation of the photochemical media in these experiments, the source of this blank may be derived from contamination from the added mediators or theoretically also from polyatomic interference ^63^Cu^40^Ar^+^ (see below). In fact, the use of sub-boil distilled water and HCOOH provided only a slight improvement in blank values and they are not essential for regular use. A slight improvement in the blank was also obtained when cobalt(II) nitrate was used instead of cobalt(II) acetate to prepare the stock solution of Co^2+^ mediator (see Supporting Information).

For comparison, LODs (3σ, *n* = 13) for conventional PN-ICP(MS)/MS employing an autosampler and continuous flow steady-state measurements were evaluated for different modes of operation of the reaction/collision cell (measurement conditions are given in Table S2). LODs corresponded to 0.025 ng L^–1^ for no gas, 0.035 ng L^–1^ for He, 0.040 ng L^–1^ for high energy He (HEHe), and 0.070 ng L^–1^ for O_2_ employing a mass-shift measurement mode (+16 *m*/*z*, ^103^Rh^+^ → ^103^Rh^16^O^+^). Despite the different means of sample introduction (FI vs continuous aspiration), it is evident that FI-PVG-ICPMS surpasses conventional PN-ICP(MS)/MS in terms of LODs by at least 2-fold, reflecting an increase in the efficiency of analyte introduction into the plasma.

### Interference Study

The effect of potential coexisting metals (i.e., Au^3+^, Fe^3+^, Mn^2+^, Pb^2+^, Pd^2+^, Pt^4+^, and Zn^2+^) that can be present in typical prepared samples of interest containing Rh (see below) was examined (Table S3). No negative interference was identified for Fe^3+^, Mn^2+^, and Zn^2+^ present in prepared samples at levels of up to 10 mg L^–1^. Lead and Pd caused a significant decrease in the peak area response (by ≈10%) at a concentration of 1 mg L^–1^. The most serious interference arises from Au^3+^ and especially Pt^4+^ ions, wherein a concentration of 0.1 mg L^–1^ in the standard solution caused a decrease in the peak area response by 50% for Pt^4+^ and 10% for Au^3+^. Measurement with 1 mg L^–1^ Au^3+^, suppressing the response to 13%, was followed by a memory effect because the response provided by the 0.5 μg L^–1^ Rh^3+^ standard without added Au^3+^ decreased by ≈15%. Since no PVG activity of Au was identified in this work, when ^197^Au was simultaneously monitored with ^103^Rh, it is likely that Au^3+^ is reduced to Au^0^ during PVG, agglomerates, and strongly deposits inside the photoreactor. To restore the PVG efficiency for Rh, the photoreactor had to be cleaned using aqua regia.

Interference effects from inorganic acids, anions, and hydrogen peroxide were also investigated. Consideration of their possible presence in samples of interest arises from their use during digestion or stabilization of samples. The effect of NO_3_^–^ has already been discussed in the Effect of Nitrates section, and at the selected optimum PVG conditions utilizing the addition of 50 mM NaNO_3_ to both sample and photochemical medium, the method still appears to be sufficiently tolerant toward additional NO_3_^–^ (at least 50 mM; see Figure 3).

Figure S7 shows that a serious suppressive effect occurs for Cl^–^ and Br^–^ because concentrations of 10 mM cause a decrease in peak area response by 25 and 60%, respectively. Such low tolerance to Cl^–^ is out of line with the PVG characteristics of other transition metals^15,16,21,22,45^ and could pose a problem when HCl is used for the preparation of real samples. The tolerance toward H_2_SO_4_ (and Na_2_SO_4_) is more than 1 order of magnitude better than for HCl/NaCl/NaBr, as a significant decrease in peak area response occurs at concentrations of 300 mM. Sodium nitrite interferes at ≥30 mM, but as noted earlier, mainly due to a physical effect related to its decomposition, i.e., bubble formation and the inability to introduce the full volume of sample into the photoreactor.

The addition of H_2_O_2_ was reported to enhance the yield of CO during irradiation of HCOOH at 254 nm,^50^ which might be useful for the synthesis of the volatile carbonyl of Rh (presumably Rh(CO)_4_H). Hence, the effect of H_2_O_2_ was also examined but absolutely no increase in the response was identified up to a concentration of 30 mM, beyond which higher concentrations led to a significant decrease in PVG efficiency (Figure S7).

### Method Validation

There is a limited choice of CRMs available with certified Rh concentrations, and no materials are available to match low Rh concentrations typically found in the environment, because it is usually present below detection capabilities of the majority of analytical methods. Since Rh is a common component of auto catalytic converters, SRM 2556 (Used Auto Catalyst) was chosen to validate the accuracy of the developed methodology. In addition, OREAS 684 (Platinum Group Element Ore) was also examined.

Metallic Rh is quite resistant to attack by all acids and low Rh recoveries from aqua regia-based digestion procedures for SRM 2556 were reported.^41^ OREAS 684 and SRM 2556 were thus prepared using a Na_2_O_2_ fusion at 650 °C and the resulting vitreous sample masses were dissolved in dilute HNO_3_ (see the Experimental Section). For analysis by FI-PVG-ICPMS, the first aliquot of the digests was prepared in a photochemical medium containing Cu^2+^ and Co^2+^ mediators. Due to the high Rh content, especially in SRM 2556, the digests were significantly diluted (200 and 4000-fold, respectively) to fit within the employed calibration range and contained approximately 6 and 50 ng L^–1^ Rh, respectively. As a result, 50 mM NaNO_3_ still had to be added to the sample because the required amount of 50 mM NO_3_^–^ was not sufficiently covered by the amount of HNO_3_ used to dissolve the fused samples. The Rh content in the digests of OREAS 684 and SRM 2556 was quantified using a standard addition technique and corrected for the total digestion blank. Spike recoveries (105 ± 2 and 100 ± 2%, respectively) of Rh standards added to the diluted digests suggest that external calibration can be reliably used for quantification at such concentration levels. A second aliquot of each sample digest was prepared in 2% (w/v) HNO_3_ and subjected to determination by conventional PN-ICP(MS)/MS, choosing He and O_2_ in the reaction/collision cell (conditions given in Table S2). The results, summarized in Table 1, indicate very good agreement between the methods as well as with the certified values.

**Table 1:** Determined Rh Concentrations (in mg kg^–1^) in CRMs by FI-PVG-ICPMS and PN-ICP(MS)/MS with He or O_2_ in the Reaction/Collision Cell

	FI-PVG-ICPMS	PN-ICP(MS)/MS		
samples	He (single quad)	He (single quad)	O_2_ (MS/MS)	certified values
OREAS 684	0.284 ± 0.005	0.286 ± 0.007	0.286 ± 0.017	0.280 ± 0.013
SRM 2556	50.4 ± 0.6	49.4 ± 0.7	49.8 ± 0.4	51.2 ± 0.5

### Analytical Application at Ultratrace Levels

Regarding potential analytical PVG applications conducted at ultratrace levels, significant concerns may arise with the use of Cu as the mediator in that its efficient PVG could result in a significant contribution to measured ^103^Rh due to a polyatomic interference (^63^Cu^40^Ar^+^) despite the use of the He collision mode. PVG efficiency of Cu was estimated using an approach similar to that used for Rh, i.e., from the comparison of the peak area of 10 mg L^–1^ Cu measured by FI-PVG and the peak area of 1 μg L^–1^ Cu in 2% (w/v) HNO_3_ measured by FI-PN (^63^Cu monitored), both processed under the same plasma conditions. PVG efficiency was very low (≈0.000052%). It agrees with a low blank response detected in the presence of both mediators and the assumption that volatile Cu species are difficult to generate.^51^ All of these refute any significant contribution to the measured ^103^Rh. For comparison, the addition of the Co^2+^ mediator results in cogeneration of volatile Co(CO)_4_H species,^52,53^ wherein the PVG efficiency at 5 mg L^–1^ was estimated to be ≈3.5%. (Note: the PVG efficiency of Co is suppressed by a factor of 2 when 50 mM NaNO_3_ is present in the photochemical medium and sample compared to PVG in the absence of NaNO_3_).

From a practical point of view, a direct analysis of two real water samples (the Vltava river and alpine lake) and CRMs (AQUA-1 and SLRS-6, not certified for Rh) was attempted to obtain some insight into the typical (natural) concentration levels of dissolved Rh. The samples were prepared with minimal dilution caused by the addition of concentrated HCOOH and solutions of NaNO_3_ and mediators, corresponding to a dilution factor of 1.75. The concentration of NO_3_^–^ in surface and ground waters typically does not exceed 10 and 50 mg L^–1^,^54,55^ i.e., 0.16 and 0.81 mM, which is negligible compared to 50 mM NaNO_3_ in the photochemical medium. The water CRMs are stabilized and contain approximately 25 mM HNO_3_ (pH 1.6), therefore, the amount of added NaNO_3_ was reduced proportionally to achieve the required concentration of 50 mM NO_3_^–^. The prepared water samples were then analyzed by FI-PVG-ICPMS using a standard addition technique, and the spike recoveries were calculated from the ratio of slopes of the standard additions (see the footnote to Table 2 for the concentration ranges) versus external calibration functions (0, 0.5, 1, and 2 ng L^–1^ standards). In parallel, the determination of Rh was conducted by conventional PN-ICP(MS)/MS employing various reaction/collision cell gas chemistry (see Table S2 for the ICP(MS)/MS setting). Results obtained by FI-PVG-ICPMS (Table 2) confirmed undetectable levels (<0.022 ng L^–1^) of dissolved Rh in AQUA-1, river, and lake water, while the Rh concentration for SLRS-6 was below the estimated LOQ (0.074 ng L^–1^). Conversely, the values obtained by PN-ICP(MS)/MS were substantially higher for all modes of the reaction/collision cell—in single digits of ng L^–1^ for no gas mode, 0.2–0.7 ng L^–1^ for He mode, 0.04–0.3 ng L^–1^ for HEHe mode, and 0.35–2 ng L^–1^ for O_2_ mass-shift mode. Interestingly, the values for AQUA-1 and SLRS-6 in the no gas mode are not far from the indicative values reported previously as part of the interlaboratory characterization of these materials.^56,57^

**Table 2:** Comparison of Determined Rh Concentrations (in ng L^–1^) in Water Samples by FI-PVG-ICPMS and PN-ICP(MS)/MS and Spiked Recoveries (%) of FI-PVG-ICPMS

	FI-PVG-ICPMS^a^		PN-ICP(MS)/MS^b^				
samples	He (single quad)	spike recovery^c^	no gas (single quad)	He (single quad)	HEHe (single quad)	O_2_ (MS/MS)	reported values^d^
AQUA-1	<0.022	103 ± 1	0.94 ± 0.04	0.17 ± 0.03	0.04–0.13	0.35 ± 0.10	0.8 ± 0.4 (ref (56))
SLRS-6	0.022–0.074	101 ± 5	1.3 ± 0.1	0.20 ± 0.06	0.04–0.13	0.36 ± 0.09	0.69 ± 0.17 (ref (57))
							0.80 ± 0.54 (ref (56))
river water	<0.022	100 ± 1	2.5 ± 0.2	0.47 ± 0.04	0.21 ± 0.03	1.0 ± 0.1	
lake water	<0.022	99 ± 1	3.8 ± 0.1	0.74 ± 0.09	0.31 ± 0.03	2.0 ± 0.3	

a LOD and LOQ for FI-PVG-ICPMS corresponded to 0.022 and 0.074 ng L^–1^ (values corrected for a dilution factor of 1.75 introduced by the preparation of water sample in the photochemical medium containing mediators).

b LOD and LOQ obtained for various modes of PN-ICP(MS)/MS corresponded to 0.025 and 0.083 ng L^–1^ for no gas, 0.035 and 0.12 ng L^–1^ for He, 0.040 and 0.13 ng L^–1^ for HEHe, and 0.070 and 0.23 ng L^–1^ for O_2_ in the reaction/collision cell.

c Spike recovery = slope of standard additions/slope of external calibration (0, 0.5, 1, and 2 ng L^–1^) × 100 (%), 0.5 and 1 ng L^–1^ Rh spiked to AQUA-1 and SLRS-6, 1 and 2 ng L^–1^ Rh spiked to river and lake water.

d Data reported with expanded uncertainty.

The biased results obtained by PN-ICP(MS)/MS were unequivocally confirmed to originate from uncorrected polyatomic interferences (see the Supporting Information for a detailed discussion), especially from ^87^Sr^16^O^+^ and ^63^Cu^40^Ar^+^ because the concentrations of Sr and Cu in these samples were determined to contain up to 190 and 70 μg L^–1^, respectively. As noted in the Introduction, the influence of these interferences becomes especially serious at ultratrace Rh concentrations, characterized by tens to hundreds of counts per second, when the relative contribution from the interferent to the apparent analyte signal intensity is large. Although it is evident that He and HEHe modes substantially improved the selectivity of Rh determination by PN-ICPMS, the kinetic energy discrimination in the collision cell cannot be absolute due to the broadening of ion kinetic energy distribution.^58^ The LODs were also being degraded due to an unavoidable parallel reduction in analyte sensitivity. Despite the capability to handle the majority of polyatomic and doubly charged ion interferences, even O_2_ mass-shift mode (+16 *m*/*z*) does not appear to provide accurate data for Rh using PN-ICPMS/MS, especially in the presence of Sr, for which the concentrations of 112 and 190 μg L^–1^ were determined in the river and lake samples, respectively. The ^87^Sr^16^O^+^ ion formed in the plasma and selected by the first mass filter (set to 103 *m*/*z*) reacts with O_2_ in the reaction cell and contributes to the selected ^103^Rh^16^O^+^ signal as ^87^Sr^16^O^16^O^+^ (see the Supporting Information for details).

In contrast, results obtained by FI-PVG-ICPMS cannot suffer from the same spectral interferences to such a serious extent because the elements potentially contributing to the polyatomic or doubly charged interfering ions are not “active” in PVG, i.e., do not form volatile species (Sr, Rb, Y, Zr, and Na), or do so with insignificant efficiency (Cu, Pb, and Zn) under the experimental conditions used (see the Supporting Information) despite their previously reported PVG activity under other conditions.^19,23,51^ The possibility of compromised PVG results due to nonspectral interferences can be reliably dismissed because spike recoveries were close to 100% (Table 2). These features highlight the importance of the developed PVG methodology needed for the accurate determination of low levels of Rh concentrations in pristine natural samples or those only slightly contaminated.

## Conclusions

In this work, substantial progress in advancing the PVG of Rh has been made. Compared to our previous studies devoted to PVG of other PGEs, i.e., Ru and Ir,^15,16,40^ volatile species of Rh are generated with the greatest difficulty, requiring a cocktail of several components in the photochemical medium, namely HCOOH, Cu^2+^ and Co^2+^ mediators, and, unexpectedly, also NO_3_^–^, the positive effect of which has not yet been identified in PVG of any other transition metal (under reductive conditions). Nevertheless, almost 15% synthesis efficiency of a new truly volatile Rh species (based on a literature survey^59−61^ likely Rh(CO)_4_H, which should be identified in the near future) seems to be a further highlight for the scientific community, in addition to the analytical benefits. The volatile species appears to be less stable compared with other transition-metal carbonyls, especially when in contact with the liquid photochemical medium. Thus, modification of the outlet arm of the commercially available thin-film flow-through photoreactor may be beneficial in follow-up studies dedicated to PVG of new analytes that produce less stable volatile species.

Despite the necessity for the preparation of a complex photochemical medium, a sufficiently good repeatability of FI-PVG-ICPMS methodology was achieved along with excellent detection power surpassing that typical of PN-ICPMS. In fact, lower LODs for any VG approach without resorting to preconcentration have been reported only for PVG of Ir to date.^15,40^ Perhaps more importantly, PVG acts as an efficient “filter” that is highly capable of removing all spectral interferences from potentially interfering elements, including Sr, Cu, Pb, and others, particularly plaguing the determination of Rh at ultratrace levels by conventional PN-ICPMS. This has been sufficiently demonstrated by the comparative analyses of natural water samples and reference materials, for which PN-ICP(MS)/MS was shown to give positively biased concentrations of Rh due to the presence of these concomitant elements, while FI-PVG-ICPMS provides still very low concentrations of Rh (<0.1 ng L^–1^) without any related concerns for possible increases due to recent anthropogenic activities.

## Supporting Information Available

Details of instrumentation; schemes of coupling PVG to ICPMS and introduction of the Ar carrier to the output from the photoreactor; ICPMS parameters for FI-PVG and conventional PN; measurement procedure and conventions; specification of analytical standards, metal ion mediators, and other chemicals; experiments dealing with the release and transport of volatile species; effects of HCOOH concentration and sample flow rate (without and with mediators); effect of added liquid ammonia to the photochemical medium; effect of residence time of volatile species dissolved in the photochemical medium; effect of introducing air segments; details of interference study; discussions on the influence of spectral interferences and PVG mechanism (PDF)
