# Supplementary material for: Sensitive and Spectral Interference-Free Determination of Rhodium by Photochemical Vapor Generation Inductively Coupled Plasma Mass Spectrometry
Source: Anal Chem. 2025 Feb 10;97(6):3545–53. doi: 10.1021/acs.analchem.4c05921 (PMC11840798; doi:10.1021/acs.analchem.4c05921)
Supplement: Supplementary file 1 — ac4c05921_si_001.pdf [file ac4c05921_si_001.pdf]

Supporting Information

Sensitive and Spectral Interference-Free Determination of Rhodium by Photochemical Vapor Generation Inductively Coupled Plasma Mass Spectrometry

Karolína Hašlová[a,b] and Stanislav Musil[a,*]

[a] Institute of Analytical Chemistry of the Czech Academy of Sciences, Veveří 97, 602 00 Brno, Czech Republic

[b] Charles University, Faculty of Science, Department of Analytical Chemistry, Hlavova 8, 128 43 Prague, Czech Republic

* Corresponding author; E-mail: stanomusil@biomed.cas.cz (S. Musil)

TABLE OF CONTENTS

Details of instrumentation.. S-4
Figure S1. PVG arrangement for FI coupled to ICPMS with simultaneous liquid nebulization.. S-5
Figure S2. Schemes of introduction of Ar carrier to the output of a thin-film flow-through photoreactor.. S-5
Table S1. Typical ICPMS parameters for coupling with PVG... S-7
Table S2. ICPMS/MS parameters for conventional PN sample introduction........................ S-7
Measurement procedure and established conventions... S-8
Specification of analytical standards, transition metal ion mediators, and other chemicals... S-9
Experiments dealing with release and transport of volatile species.................................... S-10
Details of some experiments dealing with PVG without mediators.................................... S-10
Figure S3A,B. Influence of HCOOH concentration and sample flow rate on PVG of Rh conducted without mediators.. S-10
Figure S4. Effect of added liquid ammonia to the photochemical medium comprising 10 M HCOOH.. S-11
Additional experiments related to the effect of the residence time of volatile species in the photochemical medium.. S-12
Figure S5. Effect of additional PTFE conduit volume downstream the modified photoreactor outlet and corresponding residence time of volatile species in the photochemical medium. S-12
Figure S6A,B. Influence of HCOOH concentration and sample flow rate on PVG of Rh conducted in the photoreactor with the modified outlet and in the presence of Cu^{2+} and Co^{2+} as mediators.. S-13
Experiments dealing with the effect of introducing air segments.. S-14
Details of interference study.. S-15
Table S3. Influence of various coexisting ions on Rh response.. S-15
Figure S7. Relative effects of added inorganic acids, salts, and hydrogen peroxide on PVG of Rh.. S-16
Analytical application and discussion on the influence of spectral interferences................ S-16
Table S4. Comparison of "determined Rh concentrations" in 1 mg L^{-1} standard solutions of potential interfering elements by FI-PVG-ICPMS and PN-ICP(MS)/MS S-18
Some remarks on PVG mechanism.. S-19

Figure S8. UV-vis absorption spectra of various media prepared using DIW, 0.01 M HCOOH, or 1 M HCOOH with or without addition of Rh^{3+}.. S-20
Figure S9. Comparison of UV-vis absorption spectra of media containing 0.01 M HCOOH and 0.01 M HCOOH + 0.01 M $NaNO_3$.. S-23
References.. S-26

EXPERIMENTAL SECTION

Instrumentation. Sample solutions were introduced in a flow-injection (FI) mode into a stream of the photochemical medium fed to the photoreactor with the aid of an injection valve (0.5 mL sample volume). Delivery at an arbitrary flow rate was undertaken using a peristaltic pump (Reglo Digital, Ismatec) which was also used to evacuate waste from the gas-liquid separator (GLS). With the exception of the Tygon pump tubing, all connecting tubing was made of PTFE (1 mm i.d.), unless explicitly stated otherwise. The thin-film flow-through photoreactor was a 19W low-pressure Hg discharge lamp (Jitian Instruments Co., Beijing, China) internally fitted with three efficiently irradiated lengths of synthetic quartz tubing (1 mm i.d., total volume 0.72 mL) that are successively connected by two short quartz segments (2 mm i.d.) on either end of the photoreactor that are not efficiently irradiated. In the standard configuration (Figures S1 and S2A), the photoreactor contains two quartz tubes at both ends (2 mm i.d., 5 cm long), bent to a right angle and fused to the irradiated tubes, that serve for inlet or outlet of the liquid photochemical medium containing sample. This is followed by a threaded connector (mounted on the quartz tube), a PTFE tube (1 mm i.d., 3 cm long), and a T-piece in which a continuous flow of Ar carrier gas is introduced into the photochemical medium, supporting the release of generated volatile species and their transfer to the plastic GLS (15 mL), as described elsewhere.[1,2] In the modified configuration (Figure S2B), the side outlet arm of the photoreactor was shortened and replaced with a PTFE tube of minimum dimensions (0.5 mm i.d., 1 mm o.d., 3 cm long), wherein the extremity of this tubing was inserted approximately 2 mm into the inner irradiated channel of the photoreactor and sealed. The other end of this tubing was connected directly to the T-piece so that the photochemical medium exiting from the photoreactor could be immediately mixed with the Ar carrier stream.

No special cleaning of the thin-film flow-through photoreactor was necessary between sequential measurements and the chemifold was typically only flushed with deionized water (DIW) at the end of each measurement day. From time to time, the quartz photoreactor was manually filled with concentrated HNO_3 or reverse aqua regia via a syringe and the UV lamp was powered on to initiate decomposition of acid(s), thus facilitating dissolution and removal of any deposited (metal) impurities. This cleaning procedure was also required after the experiments with ≥100 mg L^{-1} Fe^{2+} as the mediator, which caused severe memory effects and increased blank signals.

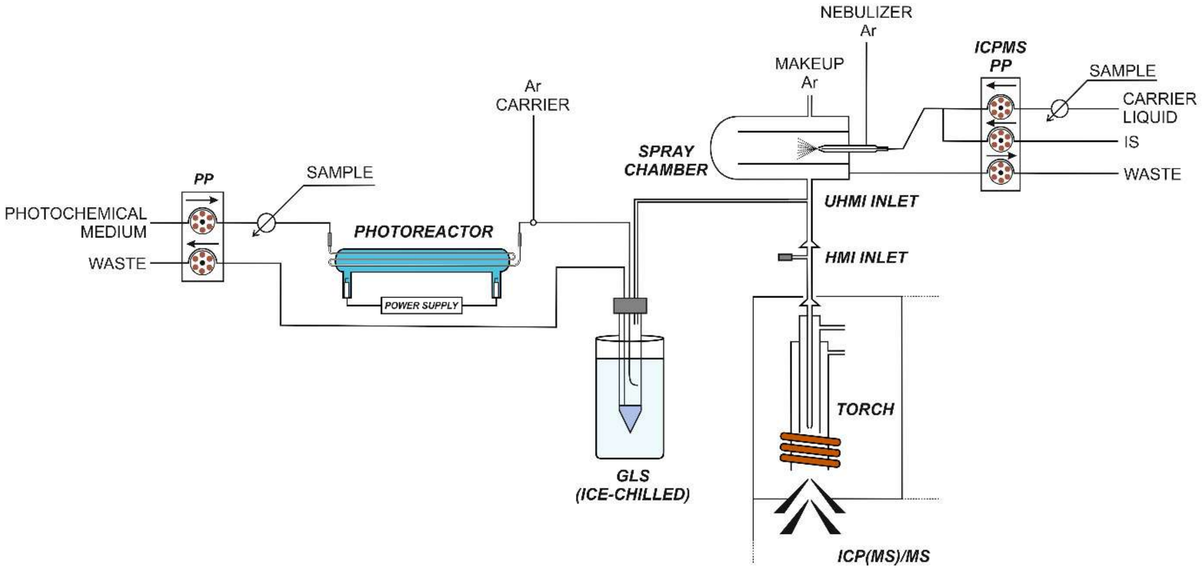

Figure S1. FI-PVG arrangement for coupling to ICPMS (Agilent 8900 ICPMS/MS in this case) with simultaneous pneumatic nebulization of liquid. GLS – gas-liquid separator, UHMI – ultra-high matrix introduction port (available with Agilent 8900 ICPMS/MS), HMI – high matrix introduction port (available with Agilent 7700x ICPMS).

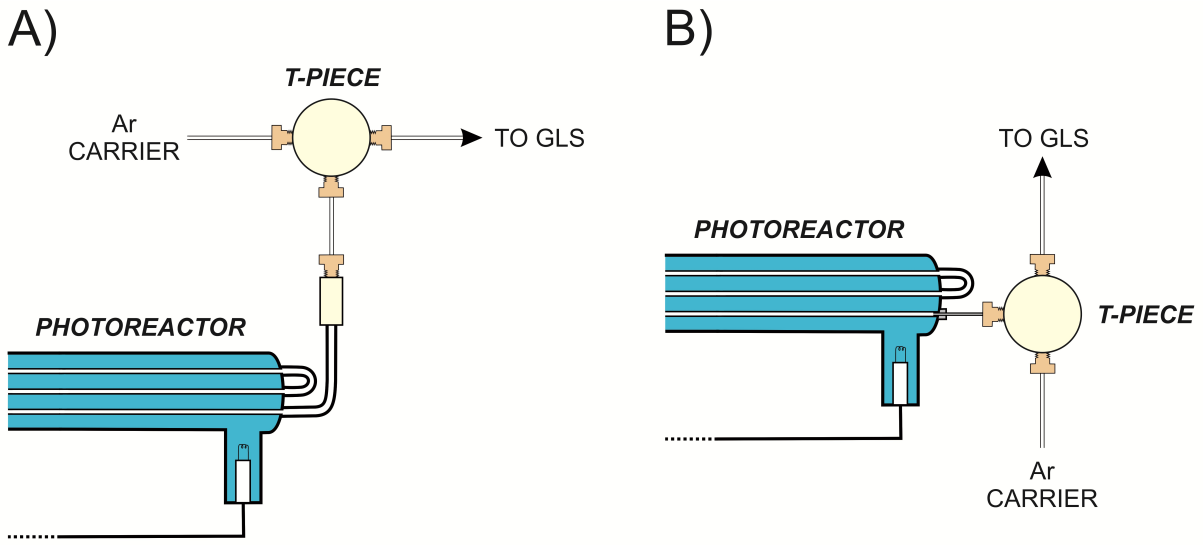

Figure S2. Schemes of introduction of Ar carrier to the output of the thin-film flow-through photoreactor, A – a standard setup, B – a modified setup enabling a fast stripping of volatile species from the photochemical medium.

In some experiments, the PVG was performed using a coiled PTFE tubing as a photoreactor. The sample solution was propelled through a PTFE tubing of 1 mm i.d. × 1.59 mm o.d. (Vici Jour Research, Switzerland) that was wrapped around the circumference of the thin-film flow-

through photoreactor. The irradiated length of the PTFE tubing was 2.5 m, resulting in approximately 2.0 mL irradiated volume. The T-piece, through which a continuous flow of Ar carrier gas was introduced into the photochemical medium, was attached to the surface of the UV lamp to minimize non-irradiated parts of the coiled photoreactor.

Detection of generated volatile Rh species was achieved using either an Agilent single quadrupole 7700x ICPMS or Agilent 8900 triple quadrupole ICPMS/MS, both employing “wet plasma" conditions created by simultaneous pneumatic nebulization (PN) of a carrier liquid (2% (w/v) HNO_3) mixed on-line with that of an internal standard (IS).[1-4] More robust conditions are created in the “wet” ICP and use of an IS permits the monitoring/correction for any sensitivity drift due to changes in the plasma or interface transmission efficiency by means of corresponding response changes noted for the nebulized IS. The outlet of the GLS was directed to the ICPMS via a PTFE tubing (2 mm i.d. x 40 cm long) through a high matrix introduction port (HMI, available with Agilent 7700x ICPMS) or ultra-high matrix introduction port (UHMI, available with Agilent 8900 ICPMS/MS), both located downstream of a Scott double-pass spray chamber (Figure S1). These ports are originally intended for on-line dilution of aerosols from samples with high salt content. Carrier liquid (2% (w/v) HNO_3), mixed with the IS solution (200 μg L^{-1} Ge and 20 μg L^{-1} In in 2% (w/v) HNO_3), was concurrently introduced into the spray chamber using a MicroMist nebulizer (Burgener Research Inc., Mississauga, Canada). The liquid carrier channel was equipped with a manual injection valve (0.5 mL sample loop volume) and it was exclusively employed to inject standards of Rh or other elements (prepared in 2% (w/v) HNO_3) to estimate/determine overall PVG efficiency (see Section “Procedure and Conventions” below).

Optimal plasma operating parameters of the Agilent 8900 ICPMS/MS for coupling with FI-PVG, monitored ions, and their dwell times are summarized in Table S1. Due to the very similar construction of the spray chamber, nebulizer, and conduit to the torch, the gas flow setting used with Agilent 7700x ICPMS was the same. Typically, isotopes of ^{103}Rh (dwell time 0.1 s), ^{72}Ge (0.05 s), and ^{115}In (0.05 s) were monitored and possibly accompanied by ^{56}Fe, ^{59}Co, ^{63}Cu, ^{66}Zn, ^{85}Rb, ^{88}Sr, ^{89}Y, ^{90}Zr, ^{197}Au, or ^{206}Pb according to the actual demands required for optimization or real sample analysis.

Table S1. Typical Agilent 8900 ICPMS/MS parameters for coupling with FI-PVG

RF power (W)	1550
RF matching (V)	1.4
Sampling depth (mm)	8.0
Nebulizer Ar (mL min^{-1})	820
Makeup Ar (mL min^{-1})	0
Ar carrier for PVG (mL min^{-1})	300
Carrier liquid/IS flows (mL min^{-1})	0.33/0.08
Spray chamber temperature (°C)	2
He collision gas flow (mL min^{-1})	4.1
Acquisition mode	Time-resolved analysis
Scan type	Single quad
Measured ions (dwell time, s)	$^{72}Ge^+$ (IS, 0.05), $^{103}Rh^+$ (0.1), $^{115}In^+$ (IS, 0.05)

Conventional PN-ICPMS/MS equipped with an Agilent SPS 4 autosampler was employed for comparative determination of Rh in real water samples, in digests of OREAS 684 (Platinum Group Element Ore) and SRM 2556 (Used Auto Catalyst) after peroxide fusion, and to evaluate LODs for comparison with those by FI-PVG-ICPMS. Five replicate steady-state measurements were acquired using a standard no gas, He, high energy He (HEHe), and O_2 in the reaction/collision cell, wherein detection in the O_2 mode was conducted using a mass-shift (+16 *m/z*). The gas flows, MS settings, and measured ions are summarized in Table S2.

Table S2. Agilent 8900 ICPMS/MS parameters for conventional PN sample introduction using various reaction/collision cell modes

	No gas	He	HEHe	O_2
RF power (W)	1550			
RF matching (V)	1.2			
Sampling depth (mm)	8.0			
Nebulizer Ar (mL min^{-1})	600			
Dilution Ar (to UHMI; mL min^{-1})	550			
Carrier liquid/IS flows (mL min^{-1})	0.33/0.08			
Spray chamber temperature (°C)	2			
Reaction/collision gas flow (mL min^{-1})	0	4.1	10	0.4
Acquisition mode	Spectrum (1 point, 5 replicates)			
Scan type	Single quad	Single quad	Single quad	MS/MS
Measured ions (dwell time, s)	$^{72}Ge^+$ (0.1), $^{103}Rh^+$ (0.5), $^{115}In^+$ (0.1), $^{185}Re^+$ (0.1)			$^{103}Rh^+ \rightarrow {}^{103}Rh^{16}O^+$ (0.5), $^{72}Ge^+ \rightarrow {}^{72}Ge^{16}O^+$ (0.1)

Procedure and Conventions. Measurements were conducted in the FI mode using a constant flow of the carrier (photochemical medium) propelled by a peristaltic pump. A standard/sample prepared in the photochemical medium and spiked with selected transition metals (as mediators), if any, was manually injected into the carrier stream at the beginning of a PVG cycle of recording signal intensities. Integration of the signal was stopped after the transient signal returned to the baseline.

Peak areas (in counts) of the FI transients, normalized to the averaged signal from a ^{72}Ge or ^{115}In as IS simultaneously admitted by PN over the same measurement time window, were employed as a measure of normalized analyte response. Each result is presented as an average of at least three peak area replicates with an uncertainty reported as ± one standard deviation (SD) or combined uncertainty where results are relative. In experiments focused on a study of the effect of various metal ion mediators and their combinations, the enhancement factor was evaluated from comparison of the peak area responses measured with metal(s) and without added metals. In a similar way, the enhancement factor was assessed for the addition of NO_3^-. Since two ICPMS instruments were used for measurements throughout the work, a normalized ^{103}Rh peak area was preferred to an absolute count value as a metric in Figures dealing with the effects of basic PVG parameters (HCOOH concentration, sample flow rate, etc.).
Overall PVG efficiency was determined according to our previously published procedure,[1-3,5-8] and is the product of an absolute nebulization efficiency and ratio of peak area sensitivities obtained with FI-PVG and FI-PN during their concurrent operation, i.e., both measured under exactly the same ICP conditions. The nebulization efficiency was determined under optimal settings of the ICPMS (Table S1) using a dynamic mass flow approach.[9]

Analytical Standards, Metal Ion Mediators, and Other Chemicals. Commercial stock analytical standard solutions of 1000 mg L^{-1} of individual elements were sourced as follows: Rh^{3+} (as $RhCl_3$) in 5% (w/w) HCl from Fluka; Fe^{3+} and Mn^{2+} in 2% (w/w) HNO_3 from Sigma-Aldrich; Cu^{2+}, Pb^{2+}, and Zn^{2+} in 2% (v/v) HNO_3 from Analytika (Czech Republic); Sr^{2+} in 2% (v/v) HCl from Analytika, Zr^{4+} in 5% (v/v) HNO_3 and 1% (v/v) HF from Analytika, Y^{3+} in 2% (v/v) HNO_3 from the Czech Metrology Institute; and Au^{3+} and Na^{+} in 1 M HCl from BDH (UK). An analytical stock solution of 1000 mg L^{-1} Rh^{3+} (as $Rh(NO_3)_3$) in 2–3% (w/w) HNO_3 from Merck was used for the comparison measurements only. In addition, a solution of 10 g L^{-1} of Pd^{2+} in 15% (w/w) HNO_3 was obtained from Sigma; a 1000 mg L^{-1} solution of Rb^{+} was prepared by dissolving solid $RbNO_3$ (Lachema, Czech Republic) in 2% (w/v) HNO_3; and a 10 g L^{-1} solution of Pt^{4+} was prepared by dissolving 0.4 g Pt sponge in 2 mL HNO_3 and 4 mL HCl at 80 °C with subsequent dilution to 40 mL by DIW.

The following compounds were used as potential metal ion mediators for PVG: cadmium(II) acetate dihydrate (p.a., Merck), cobalt(II) acetate tetrahydrate (99.999%, Alfa Aesar), copper(II) acetate (99.99%, Sigma-Aldrich), iron(II) acetate (≥99.99%, Sigma-Aldrich), manganese(II) acetate tetrahydrate (99.99%, Sigma-Aldrich), and nickel(II) acetate tetrahydrate (≥99%, Sigma-Aldrich). Stock solutions of individual metal mediators were prepared by dissolution of these metal acetates in 0.2% (v/v) CH_3COOH and contained 2.5–10 g L^{-1} of individual metals. For the evaluation of figures of merit and analytical applications, cobalt(II) nitrate hexahydrate (99.999%, Sigma-Adlrich) was dissolved in DIW and used instead of cobalt(II) acetate to prepare the 5 g L^{-1} Co^{2+} stock solution, because it provided slightly lower blank values. Since the amount of NO_3^- introduced by $Co(NO_3)_2$ was insufficient for the working standards containing 5 mg L^{-1} Co^{2+}, $NaNO_3$ had to be added separately to reach 50 mM (under optimal PVG conditions).

Other chemicals were sourced as follows: ammonium hydroxide (25%, p.a.) from Sigma-Aldrich; nitric acid (65%, semiconductor grade) and hydrochloric acid (37%, semiconductor grade) from Honeywell; acetic acid (99.8%, p.a.) and sulfuric acid (96%, chem. pure) from Lach-Ner; sodium chloride and sodium sulfate (p.a.) from Lachema; hydrogen peroxide (30%, p.a.) from Analytika; and sodium peroxide (≥95%, p.a.) from Carl Roth.

RESULTS AND DISCUSSION

Release and Transport of Volatile Species. The effect of Ar carrier flow on the release and transport of volatile species to the ICPMS was examined using 40 μg L^{-1} Rh in 10 M HCOOH as the photochemical medium and sample flow rate of 1.5 mL min^{-1}. The gas stream leaving the GLS was mixed with an additional flow of Ar (not shown in Figure S1) before it was introduced to the ICPMS. Care was taken to keep the total gas flow to the ICP the same, so as not to influence conditions in the plasma or sampling depth. The peak area response gradually increased as 50 to 300 mL min^{-1} was supplied to the GLS. At flow rates 300–600 mL min^{-1} no significant further effect of Ar carrier on PVG was observed, suggesting an efficient release of the gaseous product. A flow rate of 300 mL min^{-1} was chosen as optimal for further experiments.

PVG without Mediators. The effects of the basic PVG parameters, i.e., concentration of HCOOH and sample flow rate, influencing PVG of Rh without added transition metal ion mediators are depicted in Figure S3A,B.

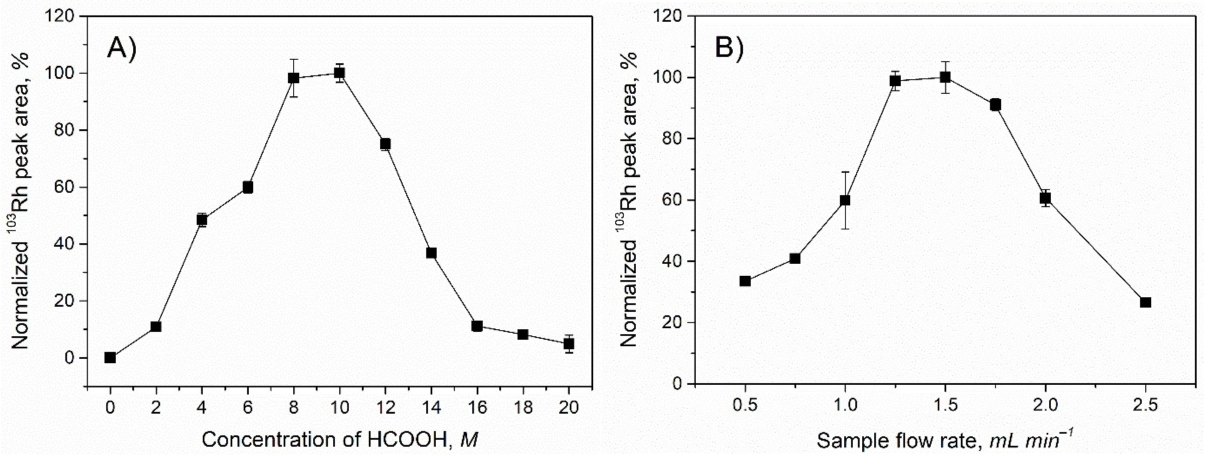

Figure S3. Influence of A) HCOOH concentration at a sample flow rate of 1.5 mL min^{-1} and B) sample flow rate at 10 M HCOOH on normalized peak area response from 40 μg L^{-1} Rh^{3+}. Peak area responses and SDs normalized to the maximum peak area value (100%).

In order to substantially enhance the overall PVG efficiency, the effect pH was also investigated as it was found useful in earlier studies for several analytes.[7,8,10-15] The effect of pH was examined by varying the volume of added liquid ammonia ($NH_3 \cdot H_2O$) to partially neutralize solutions of 10 M HCOOH (pH = 1.4). The resulting reaction media containing 0.25–4 M of formed $HCOONH_4$ in 6–9.75 M of "unreacted" HCOOH were used as the carrier and for preparation of the Rh^{3+} standard that was injected into a continuous stream of this carrier

medium. Some enhancement in PVG efficiency was identified for the photochemical media containing 0.25–1 M $HCOONH_4$, with a strong maximum at 0.5 M $HCOONH_4$ (pH = 2.5), providing a 1.7 ± 0.1-fold enhancement in the response relative to only 10 M HCOOH (Figure S4). The other media with $HCOONH_4$ concentrations ≥2 M (pH ≥ 3.1) caused a decrease in the peak area response. Due to the laborious preparation (cooling needed), risk of contamination, and especially not substantial enhancement in PVG efficiency, the use of photochemical media containing $HCOONH_4$ was not pursued further.

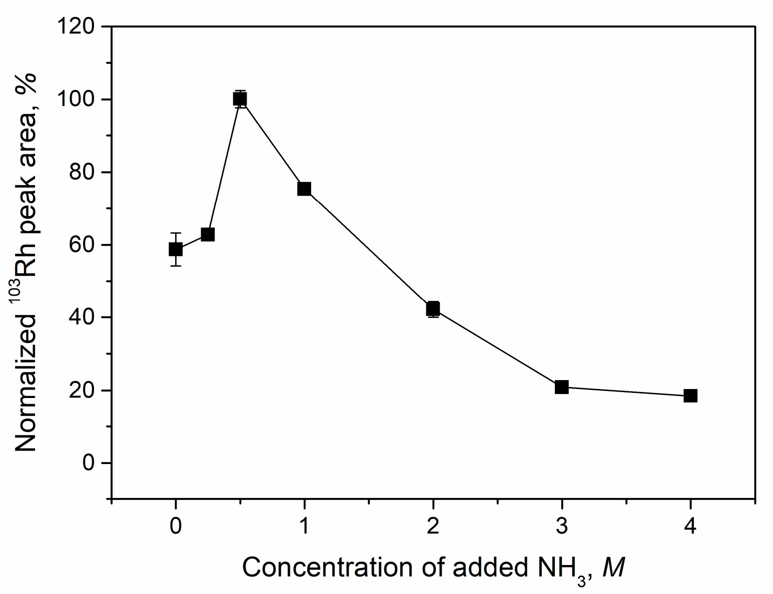

Figure S4. Effect of added liquid ammonia to the photochemical medium comprising 10 M HCOOH on normalized peak area response from 40 μg L^{-1} Rh^{3+} at a sample flow rate of 1.5 mL min^{-1}. Peak area responses and SDs normalized to the maximum peak area value (100%).

Effect of the Residence Time of Volatile Species in the Photochemical Medium. The effect of the residence time of the volatile species dissolved in the photochemical medium after exiting the photoreactor was examined by varying the length (0, 10, 20, and 30 cm) and thus the volume of a PTFE tubing (1 mm i.d.) inserted between the new exit and the T-piece for the introduction of the Ar carrier gas (Figure S5)

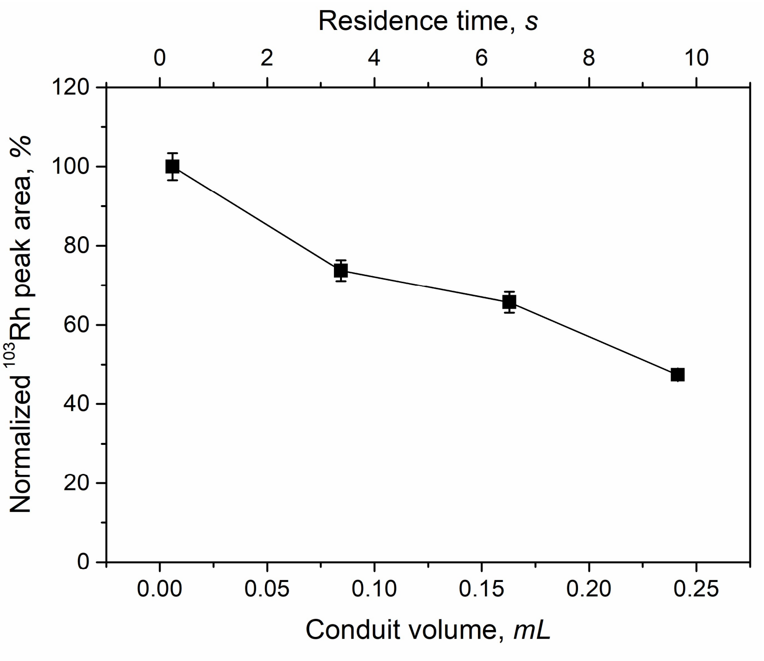

Figure S5. Effect of additional PTFE conduit volume downstream the modified photoreactor outlet (lower X axis) and corresponding residence time of volatile species in the photochemical medium (upper X axis) on normalized peak area response from 1 µg L^{-1} Rh^{3+} at a sample flow rate of 1.5 mL min^{-1}; 10 M HCOOH as the photochemical medium, 10 mg L^{-1} Cu^{2+} and 5 mg L^{-1} Co^{2+} as mediators added to the sample. Peak area responses and SDs normalized to the maximum peak area value (100%).

Due to the modification of the photoreactor/generator design, it was necessary to reinvestigate the effects of the basic PVG parameters (HCOOH concentration and sample flow rate). The dependence of peak area response on HCOOH concentration (Figure S6A) suggests that the optimum (at 10 M) was unchanged as in the case when PVG was conducted in the absence (Figure S3A) or presence of Cu^{2+} and Co^{2+} mediators (see the last paragraph in the Section ”PVG in the Presence of Metal Ion Mediators”). As for the sample flow rate, the shortening of the outlet from the photoreactor resulted in a shift of the sample flow rate providing maximum peak area response from 1.25–1.5 mL min^{-1} to 0.75–1.00 mL min^{-1} (cf. Figures S3B and S6B).

This may be a consequence of shortening the residence time of the volatile species in the photochemical medium after exiting the irradiated part of the photoreactor. Any reduction in the sample flow rate leads to a proportional extension of time before release of volatile species into the gas phase. Since the residence time in the new setup is minimal (≈0.24 s at a sample flow rate of 1.5 mL min^{-1}) compared to the original setup (≈8 s), more pronounced losses of volatile species due to chemical degradation must occur in the original setup when PVG is conducted at lower sample flow rates. Despite the maximum response, the sample flow rates of 0.75–1 mL min^{-1} are not very compatible with the FI sample introduction setup because the FI peaks became too broad, requiring long integration times and limitations on sample throughput. Therefore, 1.25 mL min^{-1} (IT = 35 s) was selected as optimum. In fact, the peak area sensitivity at this flow rate was only by 6% lower than using 1.00 mL min^{-1} that was the lowest sample flow rate characterized as providing a sufficient repeatability of measured peak areas.

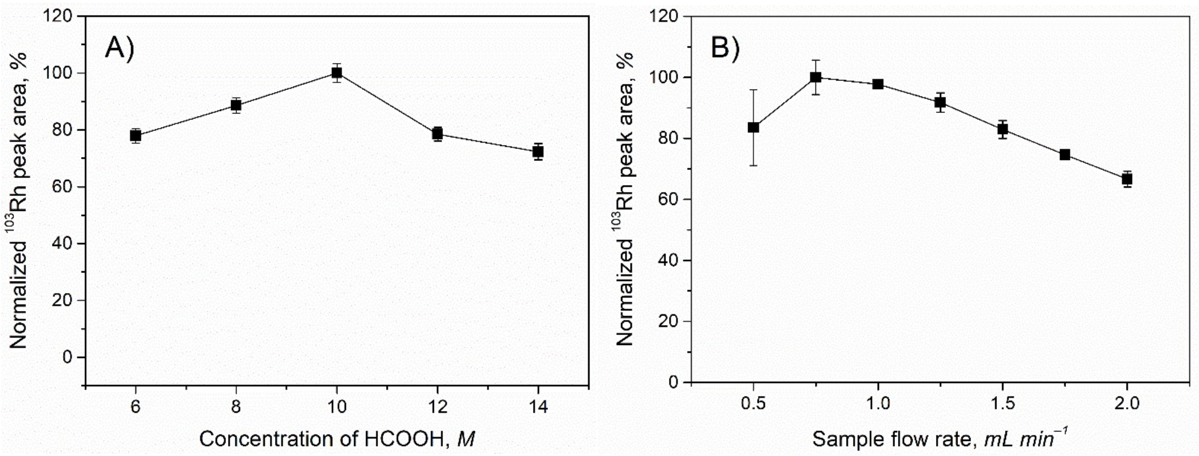

Figure S6. Influence of A) HCOOH concentration at a sample flow rate of 1.25 mL min^{-1} and B) sample flow rate at 10 M HCOOH on normalized peak area response from 1 µg L^{-1} Rh^{3+} measured with the modified photoreactor outlet; 10 mg L^{-1} Cu^{2+} and 5 mg L^{-1} Co^{2+} as mediators added to the sample. Peak area responses and SDs normalized to the maximum peak area value (100%).

Effect of Introducing Air Segments. Following earlier studies in which a significant positive effect of introducing air segments before and/or after the liquid sample zone was observed for some analytes,[16-19] we attempted to investigate this issue for PVG of Rh under chosen optimal conditions. Using our FI system for PVG, a tube for carrier was transiently removed from the vessel containing the photochemical medium (10 M HCOOH with 50 mM $NaNO_3$) while the peristaltic pump was running (at 1.25 mL min^{-1}) and an air bubble was drawn into the tube for 24 s to form an air segment in the liquid medium of 0.5 mL volume. This segment continued towards the injection valve that was manually filled with 0.25 μg L^{-1} Rh^{3+} prepared in the photochemical medium and containing 10 mg L^{-1} Cu^{2+} and 5 mg L^{-1} Co^{2+} as mediators. When approximately 0.25 mL of air segment was situated downstream of the injection valve (thus 0.25 mL was upstream of the injection valve), the sample was injected. The volume of liquid sample was thus segmented between two 0.25 mL volumes of air and continued to the photoreactor.

The measured signal intensities of ^{72}Ge and ^{115}In, used as IS, showed quite strong perturbations (stability) in the plasma due to the introduction of a small amount of O_2 in the time corresponding to the air bubbles leaving the photoreactor (i.e., before and after the measured peak). The traces of O_2 in the gas phase caused that the signal intensities of the IS to suddenly decrease sharply and then increase by about 15%, while slowly decreasing again as the liquid sample or photochemical medium exited the photoreactor. The same change in sensitivity must apply to the analyte. To obtain accurate results of the PVG efficiency enhancement, a point-by-point correction was applied to ^{103}Rh signal to compensate for such sudden changes in the IS sensitivity. Using these corrections, the increase in the PVG efficiency by introducing the air segments was 1.22 ± 0.03-fold. A comparable value was obtained when a 0.8 mL air segment (divided into 0.4 mL before and after the peak) was introduced.

This complicated measurement procedure and data evaluation were deemed not worth the small enhancement in PVG and cannot result in significantly lower LOD. In fact, such small enhancement effects from added air segments were also reported by Gao et al.[20,21] for PVG of As and Te and the authors concluded that the introduction of the air segments was not selected for further work in consideration of the analytical sensitivity and simplified operation. Therefore, this approach was not pursued further in order to keep the analytical procedure simple.

Interference Study. The effect of potential coexisting metals (i.e., Au^{3+}, Fe^{3+}, Mn^{2+}, Pb^{2+}, Pd^{2+}, Pt^{4+}, and Zn^{2+}) that can be present in typical prepared samples of interest containing Rh was examined (Table S3), employing the optimal PVG conditions (10 M HCOOH containing 50 mM $NaNO_3$ as the photochemical medium delivered at 1.25 mL min^{-1} and 10 mg L^{-1} Cu^{2+} and 5 mg L^{-1} Co^{2+} added to the sample). In addition, interference effects from inorganic acids (HCl and H_2SO_4), sodium salts (NaCl, Na_2SO_4, and $NaNO_2$), and H_2O_2 were also investigated (Figure S7).

Table S3. Influence of various concomitant ions on Rh (0.5 μg L^{-1}) response by FI-PVG-ICPMS

Concentration of interferent (mg L^{-1})	Recovery (%)[a] in the presence of the interferent						
	Au^{3+}	Fe^{3+}	Mn^{2+}	Pb^{2+}	Pd^{2+}	Pt^{4+}	Zn^{2+}
0.003	–[b]	–[b]	–[b]	–[b]	–[b]	97 ± 3	–[b]
0.01	95 ± 1	97 ± 2	98 ± 4	94 ± 3	103 ± 3	93 ± 3	99 ± 1
0.03	–[b]	–[b]	–[b]	–[b]	–[b]	85 ± 3	–[b]
0.1	90 ± 1	107 ± 3	96 ± 3	99 ± 3	96 ± 3	51 ± 2	101 ± 1
1	13 ± 1	99 ± 3	98 ± 4	88 ± 3	90 ± 2	–[b]	100 ± 1
10	–[b]	106 ± 2	95 ± 4	34 ± 1	6.6 ± 0.3	–[b]	99 ± 1

[a] uncertainty expressed as combined SD
[b] not measured

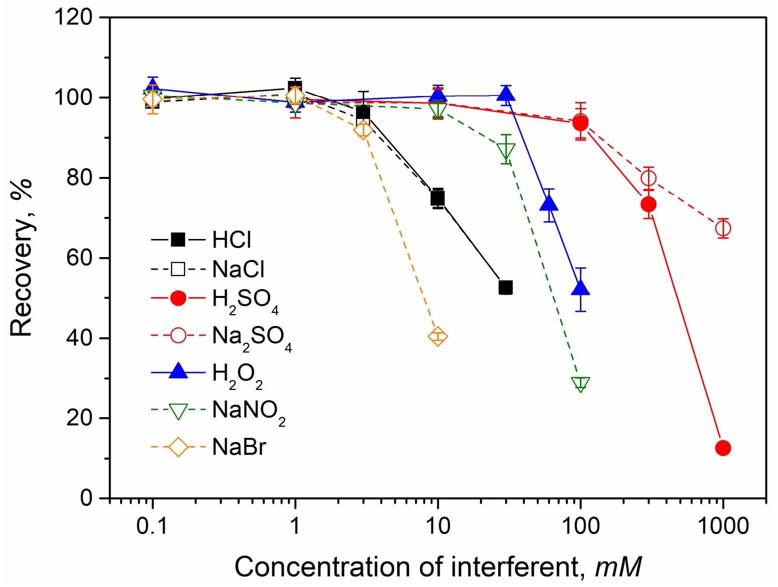

Figure S7. Relative effects of added inorganic acids, salts, and hydrogen peroxide on PVG from 0.5 μg L^{-1} Rh^{3+} prepared in 10 M HCOOH with 50 mM $NaNO_3$ and 10 mg L^{-1} Cu^{2+} and 5 mg L^{-1} Co^{2+} as mediators. Uncertainties expressed as combined SD.

Analytical Application at Ultratrace Levels. No certified or information values for Rh concentrations have been provided for AQUA-1 and SLRS-6 materials. A previous interlaboratory characterization of these materials reported indicative values of 0.8 ± 0.4 ng L^{-1} and 0.69 ± 0.17 ng L^{-1}, respectively,[22,23] but the values for SLRS-6 were based on the results provided by only one laboratory employing PN-ICPMS and no gas in the reaction/collision cell. In the last interlaboratory trial, the updated indicative value for SLRS-6 was provided (0.80 ± 0.54 ng L^{-1})[22] when it was derived from the results of two laboratories that employed PN-ICPMS with no gas in the reaction/collision cell or PN-ICPMS with a sector field analyzer and the measurement conducted at low resolution. In all cases, the values are clearly at the edge of detection capabilities. In our work, very similar values for AQUA-1 and SLRS-6 were measured by PN-ICPMS using the no gas mode (Table 2) whereas those obtained using He, HEHe, and O_2 modes were substantially lower.

Considering the various capabilities of the reaction/collision cell modes for removal of polyatomic and doubly charged ion interferences, it is evident that the results obtained with PN-ICP(MS)/MS for the two CRMs and two real samples of water (Table 2) must be biased due to uncorrected interferences from concomitant elements. Theoretically, these may be due to the presence of Cu, Sr, Rb, Pb, Zn, Y, Zr, Na, Cr, Pd, and Ru, resulting in $^{63}Cu^{40}Ar^{+}$, $^{65}Cu^{38}Ar^{+}$,

$^{87}Sr^{16}O^+$, $^{87}Rb^{16}O^+$, $^{85}Rb^{18}O^+$, $^{206}Pb^{++}$, $^{67}Zn^{36}Ar^+$, $^{66}Zn^{37}Cl^+$, $^{68}Zn^{35}Cl^+$, $^{89}Y^{14}N^+$, $^{91}Zr^{12}C^+$, $^{40}Ar^{40}Ar^{23}Na^+$, $^{53}Cr^{50}Cr^+$, $^{102}PdH^+$, and $^{102}RuH^+$ (for simplicity, only diatomic interferences and $^{40}Ar^{40}Ar^{23}Na^+$ are considered). If we narrow down the selection of potentially interfering elements according to the probability of polyatomic ion formation and the concentration (≥1 µg L^{-1}), at which the elements can be present in the water samples, we should in particular focus on Cu, Sr, Rb, Pb, and Zn, and the resulting polyatomic $^{63}Cu^{40}Ar^+$, $^{87}Sr^{16}O^+$, $^{87}Rb^{16}O^+$, and $^{67}Zn^{36}Ar^+$ and doubly charged $^{206}Pb^{++}$ ions. Based on the certificates and compiled data from the interlaboratory studies,[22,23] AQUA-1 and SLRS-6 materials contain 7.46 ± 0.12 and 23.9 ± 1.8 µg L^{-1} Cu, respectively; 36.02 ± 0.48 and 40.66 ± 0.32 µg L^{-1} Sr, respectively; 1.36 ± 0.03 and 1.41 ± 0.05 µg L^{-1} Rb, respectively; 1.364 ± 0.034 and 0.170 ± 0.026 µg L^{-1} Pb, respectively; and 0.97 ± 0.08 and 1.76 ± 0.12 µg L^{-1} Zn, respectively. As for the two real samples (the Vltava river and alpine lake), the concentrations were determined by a rapid semi-quantitative analysis accompanying the determination of Rh by PN-ICPMS using the He collision mode, wherein the certified or compiled[22] element concentrations in AQUA-1 were used for a 1-point calibration. The approximate concentrations in the river and lake samples were 1.2 and 0.07 µg L^{-1} Cu, respectively; 112 and 190 µg L^{-1} Sr, respectively; 4.6 and 0.2 µg L^{-1} Rb, respectively; <0.025 µg L^{-1} Pb in both samples; and 4.7 and 0.48 µg L^{-1} Zn, respectively. It is evident that the main source of overestimation appears to be due to the presence of Sr and possibly also Cu for which high concentrations are relevant only for AQUA-1 and SLRS-6 materials.

To confirm this possibility, the contribution of various spectral interferences arising from the potentially interfering elements was examined. The 1 mg L^{-1} standards of Cu, Sr, Rb, Pb, Zn, Y, Zr, and Na in 2% (w/v) HNO_3 were analyzed by PN-ICP(MS)/MS and a biased Rh concentration was evaluated. The results for no gas, He, HEHe, and O_2 modes are summarized in Table S4.

Table S4. Comparison of "determined Rh concentrations" (in ng L^{-1}) in 1 mg L^{-1} standard solutions of potential interfering elements by FI-PVG-ICPMS and PN-ICP(MS)/MS

Interferent	FI-PVG-ICPMS[a]	PN-ICP(MS)/MS[b]			
	He (Single quad)	No gas (Single quad)	He (Single quad)	HEHe (Single quad)	O_2 (MS/MS)
Cu	–[c]	18.9 ± 0.3	0.46 ± 0.05	<0.040	<0.070
Sr	<0.013	21.5 ± 0.7	4.1 ± 0.1	1.7 ± 0.2	9.5 ± 0.4
Rb	<0.013	0.025–0.083	<0.035	<0.040	<0.070
Pb	<0.013	19.7 ± 0.3	26.3 ± 0.6	15.3 ± 0.5	<0.070
Zn	<0.013	<0.025	<0.035	<0.040	<0.070
Y	<0.013	0.15 ± 0.02	<0.035	<0.040	<0.070
Zr	<0.013	<0.025	<0.035	<0.040	<0.070
Na	–[d]	<0.025	<0.035	<0.040	<0.070

[a] LOD and LOQ for FI-PVG-ICPMS corresponded to 0.013 and 0.042 ng L^{-1}; [b] LOD and LOQ obtained for various modes of PN-ICP(MS)/MS corresponded to 0.025 and 0.083 ng L^{-1} for no gas mode, 0.035 and 0.12 ng L^{-1} for He mode, 0.040 and 0.13 ng L^{-1} for HEHe mode, and 0.070 and 0.23 ng L^{-1} for O_2 mode; [c] not determined because 10 mg L^{-1} Cu^{2+} is added as the mediator to the sample whose contribution to measured Rh concentration is theoretically included in the measured blank response corresponding to 0.05–0.1 ng L^{-1}; [d] not determined because 50 mM $NaNO_3$ (≈1150 mg L^{-1} Na) is added to the photochemical medium and sample whose contribution to measured Rh concentration is theoretically included in the measured blank response corresponding to 0.05–0.1 ng L^{-1}

These data suggest that it may be very difficult to completely eliminate some of the interferences in PN-ICPMS/MS, namely SrO^+, while others can be completely avoided using O_2 mode (Pb^{++}). The $^{87}Sr^{16}O^+$ is formed in the plasma, selected by the first mass filter (set to 103 *m/z*), and still available for the reaction with additional O_2 in the reaction cell where it may contribute to $^{103}Rh^{16}O^+$ response as $^{87}Sr^{16}O^{16}O^+$. The on-mass measurement with O_2 in the reaction cell (both quadrupoles set to 103 *m/z*) did not provide better results because some balance between $^{87}Sr^{16}O^+$ and $^{87}Sr^{16}O^{16}O^+$ is established in the reaction cell.[24] Better results could be expected using an alternative reaction gas for PN-ICPMS/MS such as NH_3 that has been shown feasible for Rh analysis,[25,26] wherein Sr does not participate in such reactions,[24] but further investigation was beyond the scope of this work.

In contrast, results obtained by FI-PVG-ICPMS cannot suffer from such spectral interferences to such a serious extent (see Table S4) because all the elements that can give rise to any polyatomic or doubly charged ion interferences, overlapping with ^{103}Rh, were found “inactive”

in PVG, i.e., do not form volatile species at all or their PVG efficiency was found to be insignificant under the selected optimal conditions. It was demonstrated in the Section “Analytical Application at Ultratrace Levels” in the main article that the PVG efficiency of Cu is ≈0.000052%, the potential contribution of which is covered in a measured blank response corresponding to 0.05–0.1 ng L^{-1} in any case. The “PVG efficiencies” of the other potentially interfering elements were also evaluated from comparison of the FI-PVG-ICPMS peak area responses arising from 1 mg L^{-1} standard solutions (prepared in the photochemical medium containing mediators and $NaNO_3$) and responses obtained from 1 μg L^{-1} standard solutions admitted for PN-ICPMS (10 mg L^{-1} and 20 μg L^{-1} used for Zn, respectively, due to low sensitivity). The introduction efficiencies for Sr, Rb, Pb, Zn, Y, and Zr were very low and ranged from 0.000014 to 0.000017%, suggesting that this fraction is not delivered to the ICPMS as a result of a PVG activity but rather attributed to a physical transport of cogenerated fine aerosol. This is quite surprising for Pb and Zn, for which the methodologies based on PVG have been previously developed,[27,28] but is likely due to the presence of high concentration of NO_3^- employed in this photochemical medium that significantly interferes with PVG of Pb and Zn.

Some Remarks on PVG Mechanism. It is assumed that the volatile species synthesized by PVG is $Rh(CO)_4H$[29-31] based on the use of HCOOH, possibly giving rise to only hydrided/carbonylated adducts, and fulfilling the 18-valence electron rule. However, successful identification has not yet been achieved.

The mechanism of PVG likely involves reduction of Rh^{3+} followed by the rapid uptake of gaseous CO, readily available as a product of photolysis of HCOOH, and H, likely from $H^{\bullet}$ generated in the liquid photochemical medium. The crucial enhancement effects come from the synergistic impact of the added Cu^{2+} and Co^{2+} as the mediators and the presence of NO_3^-. Although the exact role of this combination of metal ion mediators remains to be fully elucidated, recent studies using an electron paramagnetic resonance (EPR) spin trapping technique support the original thesis[32] that transition metal ions enhance the yield of highly reducing $CO_2^{\bullet-}$ during UV irradiation of HCOOH and CH_3COOH media.[16,20,21,33-35] The mechanism was very recently advanced by Sturgeon et al.,[36] who provided evidence of increased production of gases generated from HCOOH and CH_3COOH media in the presence of added metal ions (namely CO, CO_2, H_2, and CH_4), i.e., some of them closely associated with the formulation of the volatile species.

The understanding of the positive effect of NO_3^- can be based on several factors, nevertheless, the fact that NO_3^- is usually a serious interferent with the PVG methodology, quenching free

radicals and $e_{(aq)}^-$ during PVG,[32,37] should not be lost sight of. The first clue may lie in a coordination sphere of Rh atom in the photochemical medium at ultratrace levels and the hypothesis[38] that some complex ions are more easily "attacked" by generated free radicals ($CO_2^{\bullet -}$ and $H^\bullet$) and $e_{(aq)}^-$ than others, i.e., Rh is more easily reduced because of the differences in their reduction potentials.[39] Rhodium is typically found in the environment in the trivalent form. In aqueous solutions, it is present as Rh^{3+}, or rather as an aquated complex ion $[Rh(H_2O)_6]^{3+}$. Depending on the composition of the photochemical medium used, substitution of the H_2O ligand(s) with others must be considered. In the solution containing 10 M HCOOH (found to be optimal for PVG), where the degree of dissociation of HCOOH is around 0.4%, the coordination sphere of the Rh atom can be modified by the incorporation of $HCOO^-$ as the ligand, or $HCOO^-$ remains outside and acts only as a counterion to stabilize the complex. To the best of our knowledge, there is a lack of information about this in the literature. As shown in Figure S8, the UV-vis spectrometry experiments also did not confirm the formation of any Rh formato complexes.

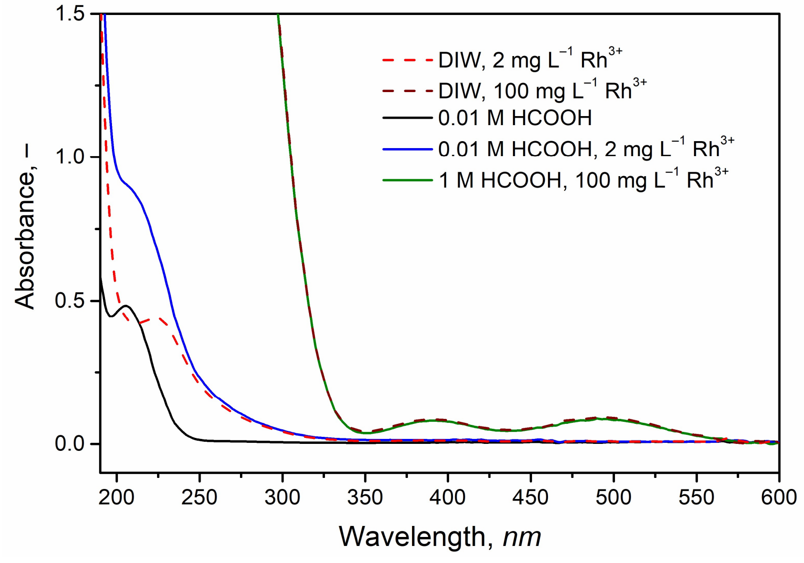

Figure S8. UV-vis absorption spectra of various media prepared using DIW, 0.01 M HCOOH, or 1 M HCOOH referenced against DIW, with or without addition of 2 mg L^{-1} or 100 mg L^{-1} Rh^{3+}.

Addition of 2 mg L^{-1} and 100 mg L^{-1} Rh^{3+} (from the stock solution of 1000 mg L^{-1} Rh in 5% (w/w) HCl ≈ 1.4 M HCl, prepared from $RhCl_3$) to DIW results in the appearance of a significant absorption band with maximum at about 220–230 nm. This band cannot be attributed to the residual HCl from the stock standard solution because no absorption band appeared in this

region when the UV-vis spectrum of 50 mM HCl in DIW (referenced against DIW) was measured. Two other bands, much weaker, were identified with 100 mg L^{-1} Rh^{3+}, with maxima at 390–395 nm and 490–500 nm. These are related to the presence of Rh aqua/chloro complexes[40-42] (see below). From our point of view, it is interesting that for 100 mg L^{-1} Rh^{3+} in 1 M HCOOH no change in the position of these two absorption bands was observed. Similarly, when the UV-vis spectrum of 2 mg L^{-1} Rh^{3+} was measured in 0.01 M HCOOH, no new absorption bands with maximum in the range 250–350 nm appeared, and this spectrum fully corresponded to the sum of the two spectra obtained with 0.01 M HCOOH and 2 mg L^{-1} Rh^{3+} in DIW.

The traces of acid used for stabilization of the stock solution of 1000 mg L^{-1} Rh, prepared from $RhCl_3$ (in our case in ≈1.4 M HCl) may play some role. Substitution of the H_2O ligand(s) by Cl^- and formation of various $[Rh(H_2O)_{6-x}Cl_x]^{3-x}$ complexes, where x = 0–6, has been documented many times[40-43] and depends on the concentration of HCl. However, in dilute HCl solutions, when the concentration of Cl^- is <1 mM, it was demonstrated that the major fraction corresponds only to Rh^{3+} (as $[Rh(H_2O)_6]^{3+}$).[40-42] This is relevant to our PVG studies because the Rh concentrations used throughout this study were ≤40 μg L^{-1} Rh^{3+}, thus resulting in ≤0.056 mM Cl^-. Similar to chlorides, the presence of added NO_3^- also significantly changes the coordination sphere of the Rh ion prior to PVG, yielding either $[Rh(H_2O)_{6-x}(NO_3)_x]^{3-x}$, where x = 1–6, wherein NO_3^- is monodentate coordinated to Rh, or $[Rh(H_2O)_{6-2x}(NO_3)_x]^{3-x}$, where x = 1–3, wherein NO_3^- is bidentate coordinated to Rh.[43-45] Nevertheless, a stable trinitrato Rh complex ($[Rh(NO_3)_3]$) preferably exists in aqueous solutions, unless another strong complexing ion is present[43] and its formation may be responsible for the enhancement in PVG efficiency. Similar to the addition of HCOOH, no such a complex was identified by the UV-vis measurements (not shown). (Note: Since the Rh absorption bands in the visible region appear to be weak, high Rh concentrations are required for UV-vis measurements but the significant amount of Cl^- and HCl from the stock standard solution is also introduced, which affects the coordination of the ligands. Therefore, for studies aimed at demonstrating the formation of specific Rh formato or nitrato complexes in the model photochemical medium, an isolation of pure and concentrated Rh hexaaqua complex ion would be appropriate.)

It is important to note that the PVG experiments performed with the working solutions prepared from the stock solutions of 1000 mg L^{-1} Rh, either from $RhCl_3$ in ≈1.4 M HCl or $Rh(NO_3)_3$ in 2–3% (w/w) HNO_3 (≈0.32–0.48 M HNO_3), showed that there was no difference between the PVG responses using various PVG conditions (namely 10 M HCOOH without added metal ion

mediators to the sample and with the addition of 10 mg L^{-1} Cu^{2+} and 5 mg L^{-1} Co^{2+}, or the optimal composition based on 10 M HCOOH + 50 mM $NaNO_3$ in the photochemical medium and the addition of 10 mg L^{-1} Cu^{2+} and 5 mg L^{-1} Co^{2+}). These experiments clearly proved that the effect of traces of acids from the stock standard solutions on PVG efficiency, and likely its mechanism, can be safely ruled out. Also, the effect of NO_3^- was not associated with the presence of Cu^{2+} and Co^{2+} mediators because it was also identified when no mediators were added to the sample and PVG was conducted only from HCOOH.

The hypothesis that some Rh complexes can be more easily converted to volatile species during PVG than others can be supported by the results obtained from the interference study. The serious interference from Cl^- is already evident at 10 mM levels (Figure S7). This may suggest a substitution of H_2O ligand(s) by Cl^- and formation of various Rh aqua/chloro complexes, depending on the concentration of Cl^-, as described previously.[40-42] This is supported by the similar or even stronger interference of Br^- (Figure S7), the presence of which results in a formation of various $[Rh(H_2O)_{6-x}Br_x]^{3-x}$ complexes, again depending on the concentration of Br^-.[46] The observed strong halide interferences on PVG of Rh are not consistent with the interfering effect of Cl^- observed for PVG of Ir,[7] i.e., a very similar analyte to Rh (group 9 of the periodic table), the PVG of which probably results in a structurally similar volatile compound, i.e., $Ir(CO)_4H$. In contrast to Rh, Ir showed a very good tolerance towards Cl^- (up to 1 M). However, this discrepancy could be explained by the much greater inertness of $[Ir(H_2O)_6]^{3+}$ to attack by Cl^- compared to $[Rh(H_2O)_6]^{3+}$.[47]

All of the above speculations were derived from the effects occurring in the liquid media prior to PVG. However, NO_3^- can also participate in the PVG process itself and, as noted above, it is usually a serious interferent with the PVG methodology, quenching free radicals and $e_{(aq)}^-$ during generation.[32,37,48] Nitrates are reduced to NO_2^- during PVG, which continues to produce NO_2 and NO,[32,49] wherein NO_2^- was shown to be a more serious interferent with PVG of Se conducted from HCOOH and CH_3COOH media.[49,50] Conversely, in PVG of Mo conducted in HCOOH without added metal ion mediators, it was shown to interfere at approximately one order of magnitude higher concentrations than NO_3^-.[4] The experiments with added $NaNO_2$ (Figure 3) revealed no positive effect on PVG of Rh, which means that the enhancement must be attributed to only NO_3^- and not to the species resulting from photolysis of NO_3^- during PVG. The negative effect of NO_2^- at 50 and 100 mM cannot be directly attributed to chemical interference of this anion on PVG because samples with such NO_2^- concentrations were quickly saturated with gases by decomposition of $NaNO_2$ making it impossible to deliver a full volume

of sample (0.5 mL) to the photoreactor due to many bubbles formed in the sample loop of the injection valve.

The presence of NO_3^- may also alter the identity of the generated volatile species as shown for PVG of Se.[50-52] While a mixture of both SeH_2 and SeCO was identified in the gas phase when the PVG was performed in 0.7 M HCOOH, the addition of 10 mM $NaNO_3$ to this photochemical medium resulted in enhanced production of SeCO while completely suppressing the formation of SeH_2. Similar changes in the identity of the volatile product of Rh cannot be excluded. This hypothesis could be further supported by the existence of volatile $Rh_2(CO)_4Cl_2$,[29] the formation of which would require Cl^- to be incorporated into the Rh carbonyl structure (instead of H) during PVG in the Cl^- containing photochemical medium. Although this compound was also described as volatile, its stability and volatility/solubility may differ significantly from the original product and may be responsible for the observed interferences from Cl^-. The unambiguous identification of the volatile species generated in the photochemical media with and without NO_3^- (or Cl^-) could help to clarify this issue.

The last effect of added NO_3^- lies in the change of the absorption characteristic of the photochemical medium, as evident in Figure S9.

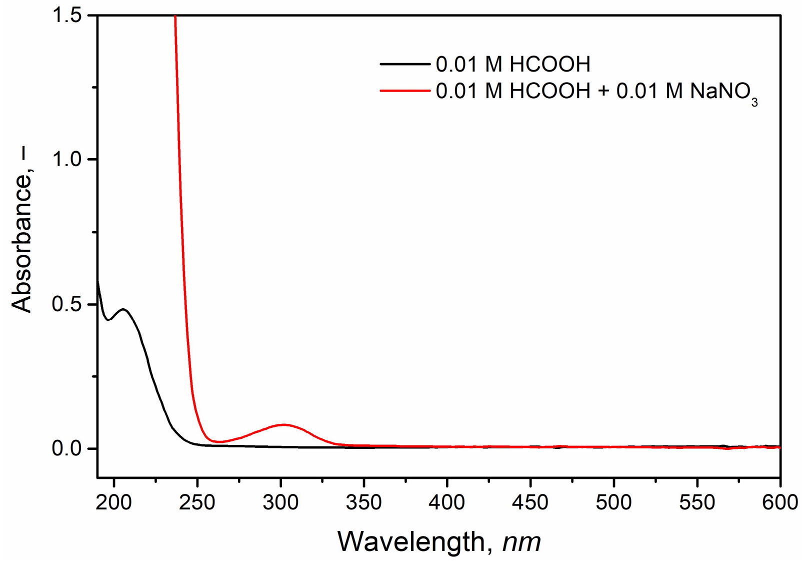

Figure S9. Comparison of UV-vis absorption spectra of media containing 0.01 M HCOOH and 0.01 M HCOOH + 0.01 M $NaNO_3$ referenced against DIW.

The spectrum exhibits a specific absorption band for NO_3^- with a maximum at around 305 nm.[53] More importantly, NO_3^- strongly adsorbs at the 185 nm line available in the thin-film flow-

through photoreactor, wherein the molecular absorption coefficient of HNO_3 at 185 nm is between 4700 and 5700 M^{-1} cm^{-1} at 25 °C,[54,55] compared to 34.9 M^{-1} cm^{-1} for undissociated HCOOH.[56] This means that the absorption coefficient of NO_3^- at 185 nm is 135–163-fold greater than that of HCOOH. Considering 10 M HCOOH and the addition of 50 mM $NaNO_3$ (i.e., 200-fold molar excess of HCOOH over $NaNO_3$), the contribution of NO_3^- to the absorption of 185 nm photon flux remains significant and provides for a lower photon penetration depth into the liquid photochemical medium and greater shadowing effect that may protect the unstable volatile analyte species from excessive UV.[6] The rate of HCOOH decomposition is also attenuated by the same effect, in addition to the terminating reactions of NO_3^- with free radicals and $e_{(aq)}^-$. These changes in the physical properties of the liquid medium due to the addition of NO_3^- may also be part of the reason for the decrease in CO and H_2 gas formation as recently demonstrated by Sturgeon et al.[36]

To gain further insight into the real effect of UV wavelength, the PVG of Rh was performed using the same thin-film flow through-photoreactor, but its circumference was wrapped with a PTFE tubing, in which the sample was propelled and irradiated. This arrangement does not allow the analyte solution to be exposed to 185 nm radiation.[57,58] The transmittance of 254 nm radiation is also significantly reduced when using this coiled photoreactor, for the ≈0.3 mm PTFE wall thickness, to approximately 52% of that of the synthetic quartz tube.[59] In addition, the inner quartz channels in the thin-film flow-through photoreactor are immersed in the discharge and thus more efficiently irradiated from all directions. Therefore, to provide a fair comparison of the performance, an irradiation time (IT) in the PTFE coiled photoreactor had to be optimized. Using 10 M HCOOH and 50 mM $NaNO_3$ in the photochemical medium and the addition of 10 mg L^{-1} Cu^{2+} and 5 mg L^{-1} Co^{2+} to the sample, as found to be optimal for the thin-film flow-through photoreactor, the highest PVG efficiency was achieved with a 2.5 m length of the PTFE tubing and a sample flow rate of 2.5–3 mL min^{-1}, corresponding to the IT of 39–47 s. Despite the significant changes in irradiation characteristics, these ITs are well comparable to the optimal IT selected for the thin-film flow-through photoreactor (approximately 35 s). More importantly, the PVG efficiency achieved with the PTFE coiled photoreactor was 46% of that achieved with the thin-film flow-through photoreactor. In the light of our previous studies, this relative value and corresponding absolute PVG efficiency (≈6.7%) seem really high, because almost no or significantly lower relative responses were obtained for PVG of Mo, W, and Ru carried out with the PTFE coiled photoreactor.[1,3,4] This is evidence that 185 nm radiation is not critical for PVG of Rh. For the addition of Cu^{2+} and the mixture of Cu^{2+} and Co^{2+} as the

mediators, a similar trend in the enhancement of the PVG efficiency was observed as for the thin-film flow-through photoreactor, although the individual values of the enhancement factor were different (PVG efficiencies were ≈0.001%, ≈0.3%, and 1.5% using 10 M HCOOH without mediators, with 10 mg L^{-1} Cu^{2+}, and with 10 mg L^{-1} Cu^{2+} and 5 mg L^{-1} Co^{2+}, respectively). This observation indicates that the positive effect of the metal mediators is not only associated with the 185 nm radiation and it occurs when PVG of Rh is conducted in the photoreactor with an access only to 254 nm radiation. Finally, the effect of NO_3^- was examined in the PTFE coiled photoreactor. The analytical responses obtained during PVG with Cu^{2+} and Co^{2+} mediators were compared in the absence or in the presence of 50 mM $NaNO_3$ in the photochemical medium. The enhancement factor was approximately 4.5, higher than that observed using the thin-film flow-through photoreactor (≈2.2, see Figure 3). However, regardless of the reason for this higher factor, it is clear that the beneficial effect of $NaNO_3$ cannot be attributed to the changes in the absorption characteristics of the liquid photochemical medium and the greater shadowing effect that protects the volatile analyte species from excessive 185 nm radiation.

In summary, there are many potential effects of NO_3^- that may give rise to enhanced PVG of Rh but with current knowledge there appears to be no definitive explanation. In our opinion, the most plausible explanation is based on the modification of the coordination sphere of Rh in the photochemical medium by added NO_3^-. A study of a change in the amount of generated $CO_2^{\bullet-}$ using electron paramagnetic resonance spin trapping techniques,[16,33-35] but also $e_{(aq)}^-$ and $H^{\bullet}$, would be interesting; unfortunately, we cannot realize this measurement now due to a lack of suitable instrumentation. Convincing identification of the generated volatile species of Rh is also mandatory.

REFERENCES

(1) Vyhnanovský, J.; Sturgeon, R. E.; Musil, S. Cadmium Assisted Photochemical Vapor Generation of Tungsten for ICPMS detection. *Anal. Chem.* **2019**, *91*, 13306–13312.

(2) Vyhnanovský, J.; Yildiz, D.; Štádlerová, B.; Musil, S. Efficient photochemical vapor generation of bismuth using a coiled Teflon reactor: Effect of metal sensitizers and analytical performance with flame-in-gas-shield atomizer and atomic fluorescence spectrometry. *Microchem. J.* **2021**, *164*, No. 105997.

(3) Musil, S.; Vyhnanovský, J.; Sturgeon, R. E. Ultrasensitive Detection of Ruthenium by Coupling Cobalt and Cadmium Ion-Assisted Photochemical Vapor Generation to Inductively Coupled Plasma Mass Spectrometry. *Anal. Chem.* **2021**, *93*, 16543–16551.

(4) Šoukal, J.; Sturgeon, R. E.; Musil, S. Efficient Photochemical Vapor Generation of Molybdenum for ICPMS Detection. *Anal. Chem.* **2018**, *90*, 11688–11695.

(5) Jeníková, E.; Nováková, E.; Hraníček, J.; Musil, S. Ultra-sensitive speciation analysis of tellurium by manganese and iron assisted photochemical vapor generation coupled to ICP-MS/MS. *Anal. Chim. Acta* **2022**, *1201*, No. 339634.

(6) Jeníková, E.; Vyhnanovský, J.; Hašlová, K.; Sturgeon, R. E.; Musil, S. Efficient Photochemical Vapor Generation from Low Concentration Formic Acid Media. *Anal. Chem.* **2024**, *96*, 1241–1250.

(7) Musil, S.; Jeníková, E.; Vyhnanovský, J.; Sturgeon, R. E. Highly Efficient Photochemical Vapor Generation for Sensitive Determination of Iridium by Inductively Coupled Plasma Mass Spectrometry. *Anal. Chem.* **2023**, *95*, 3694–3702.

(8) Nováková, E.; Horová, K.; Červený, V.; Hraníček, J.; Musil, S. UV photochemical vapor generation of Cd from a formic acid based medium: optimization, efficiency and interferences. *J. Anal. At. Spectrom.* **2020**, *35*, 1380–1388.

(9) Cuello-Nuñez, S.; Abad-Álvaro, I.; Bartczak, D.; Busto, M. E. D.; Ramsay, D. A.; Pellegrino, F.; Goenaga-Infante, H. The accurate determination of number concentration of inorganic nanoparticles using spICP-MS with the dynamic mass flow approach. *J. Anal. At. Spectrom.* **2020**, *35*, 1832–1839.

(10) de Jesus, C. H.; Grinberg, P.; Sturgeon, R. E. System optimization for determination of cobalt in biological samples by ICP-OES using photochemical vapor generation. *J. Anal. At. Spectrom.* **2016**, *31*, 1590–1604.

(11) Deng, H.; Zheng, C.; Liu, L.; Wu, L.; Hou, X.; Lv, Y. Photochemical vapor generation of carbonyl for ultrasensitive atomic fluorescence spectrometric determination of cobalt. *Microchem. J.* **2010**, *96*, 277–282.

(12) Guo, X.; Sturgeon, R. E.; Mester, Z.; Gardner, G. UV photosynthesis of nickel carbonyl. *Appl. Organomet. Chem.* **2004**, *18*, 205–211.

(13) Šoukal, J.; Musil, S. Detailed evaluation of conditions of photochemical vapor generation for sensitive determination of nickel in water samples by ICP-MS detection. *Microchem. J.* **2022**, *172*, No. 106963.

(14) Zheng, C.; Sturgeon, R. E.; Brophy, C.; Hou, X. Versatile Thin-Film Reactor for Photochemical Vapor Generation. *Anal. Chem.* **2010**, *82*, 3086–3093.

(15) Zheng, C.; Sturgeon, R. E.; Brophy, C. S.; He, S.; Hou, X. High-Yield UV-Photochemical Vapor Generation of Iron for Sample Introduction with Inductively Coupled Plasma Optical Emission Spectrometry. *Anal. Chem.* **2010**, *82*, 2996–3001.

(16) Yu, Y.; Chen, H.; Zhao, Q.; Mou, Q.; Dong, L.; Wang, R.; Shi, Z.; Gao, Y. Impact of Gas-Liquid Interface on Photochemical Vapor Generation. *Anal. Chem.* **2021**, *93*, 3343–3352.

(17) Dong, L.; Wang, W.; Ning, Y.; Deng, X.; Gao, Y. Detection of trace antimony by vanadium (IV) ion assisted photochemical vapor generation with inductively coupled plasma mass spectrometry measurement. *Anal. Chim. Acta* **2023**, *1251*, No. 341006.

(18) Yu, Y.; Hu, J.; Zhao, X.; Liu, J.; Gao, Y. Photochemical vapor generation for germanium: synergistic effect from cobalt/chloride ions and air-liquid interfaces. *Anal. Bioanal. Chem.* **2022**, *414*, 5709–5717.

(19) Bao, H.; Peng, X.; Song, Z.; Ning, Y.; Yu, Y.; Gao, Y. Natural mineral assisted photochemical vapor generation for determination of trace inorganic arsenic by inductively coupled plasma mass spectrometry. *Microchem. J.* **2021**, *170*, No. 106689.

(20) Deng, X.; Dong, L.; Chen, H.; Wang, W.; Yu, Y.; Gao, Y. Sensitive Determination of Arsenic by Photochemical Vapor Generation with Inductively Coupled Plasma Mass Spectrometry: Synergistic Effect from Antimony and Cadmium. *Anal. Chem.* **2024**, *96*, 652–660.

(21) Dong, L.; Chen, H.; Ning, Y.; He, Y.; Yu, Y.; Gao, Y. Vanadium Species-Assisted Photochemical Vapor Generation for Direct Detection of Trace Tellurium with Inductively Coupled Plasma Mass Spectrometry. *Anal. Chem.* **2022**, *94*, 4770–4778.

(22) Yeghicheyan, D.; Grinberg, P.; Alleman, L. Y.; Belhadj, M.; Causse, L.; Chmeleff, J.; Cordier, L.; Djouraev, I.; Dumoulin, D.; Dumont, J.; et al. Collaborative determination of trace

element mass fractions and isotope ratios in AQUA-1 drinking water certified reference material. *Anal. Bioanal. Chem.* **2021**, *413*, 4959–4978.

(23) Yeghicheyan, D.; Aubert, D.; Bouhnik-Le Coz, M.; Chmeleff, J.; Delpoux, S.; Djouraev, I.; Granier, G.; Lacan, F.; Piro, J.-L.; Rousseau, T.; et al. A New Interlaboratory Characterisation of Silicon, Rare Earth Elements and Twenty-Two Other Trace Element Concentrations in the Natural River Water Certified Reference Material SLRS-6 (NRC-CNRC). *Geostand. Geoanal. Res.* **2019**, *43*, 475–496.

(24) Sugiyama, N.; Nakano, K. *Reaction data for 70 elements using O_2, NH_3 and H_2 gases with the Agilent 8800 Triple Quadrupole ICP-MS. Agilent Technical Note.*; Agilent Technologies, Inc. 2014. https://www.agilent.com/cs/library/technicaloverviews/public/5991-4585EN_TechNote8800_ICP-QQQ_reactiondata.pdf (accessed January 04, 2025).

(25) Suoranta, T.; Bokhari, S. N. H.; Meisel, T.; Niemelä, M.; Perämäki, P. Elimination of Interferences in the Determination of Palladium, Platinum and Rhodium Mass Fractions in Moss Samples using ICP-MS/MS. *Geostand. Geoanal. Res.* **2016**, *40*, 559–569.

(26) Sugiyama, N.; Shikamori, Y. Removal of spectral interferences on noble metal elements using MS/MS reaction cell mode of a triple quadrupole ICP-MS. *J. Anal. At. Spectrom.* **2015**, *30*, 2481–2487.

(27) Gao, Y.; Xu, M.; Sturgeon, R. E.; Mester, Z.; Shi, Z.; Galea, R.; Saull, P.; Yang, L. Metal Ion-Assisted Photochemical Vapor Generation for the Determination of Lead in Environmental Samples by Multicollector-ICPMS. *Anal. Chem.* **2015**, *87*, 4495–4502.

(28) Hu, J.; Yu, Y.; Xiao, Z.; Gao, Y. Photochemical vapor generation of Zinc and Gallium. *Microchem. J.* **2023**, *193*, No. 109178.

(29) Hieber, W.; Lagally, H. Über Metallcarbonyle. XLV. Das Rhodium im System der Metallcarbonyle. *Z. Anorg. Allg. Chem.* **1943**, *251*, 96–113.

(30) Li, C.; Widjaja, E.; Chew, W.; Garland, M. Rhodium tetracarbonyl hydride: the elusive metal carbonyl hydride. *Angew. Chem.* **2002**, *114*, 3939–3943.

(31) Vidal, J. L.; Walker, W. E. Rhodium Carbonyl Cluster Chemistry under High Pressure of Carbon Monoxide and Hydrogen. 3. Synthesis, Characterization, and Reactivity of HRh(CO)4. *Inorg. Chem.* **1981**, *20*, 249–254.

(32) Sturgeon, R. E. Photochemical vapor generation: a radical approach to analyte introduction for atomic spectrometry. *J. Anal. At. Spectrom.* **2017**, *32*, 2319–2340.

(33) Zeng, W.; Hu, J.; Chen, H.; Zou, Z.; Hou, X.; Jiang, X. Cobalt ion-enhanced photochemical vapor generation in a mixed acid medium for sensitive detection of tellurium(iv) by atomic fluorescence spectrometry. *J. Anal. At. Spectrom.* **2020**, *35*, 1405–1411.

(34) Zhen, Y.; Chen, H.; Zhang, M.; Hu, J.; Hou, X. Cadmium and cobalt ions enhanced-photochemical vapor generation for determination of trace rhenium by ICP-MS. *Appl. Spectrosc. Rev.* **2022**, *57*, 318–337.

(35) Hu, J.; Chen, H.; Hou, X.; Jiang, X. Cobalt and Copper Ions Synergistically Enhanced Photochemical Vapor Generation of Molybdenum: Mechanism Study and Analysis of Water Samples. *Anal. Chem.* **2019**, *91*, 5938–5944.

(36) Sturgeon, R. E.; Pagliano, E.; Lopes, G. S.; Neto, R. S. A.; Brito, J. K. S. Insights into the role of transition and noble metals mediating photochemical vapor generation. *J. Anal. At. Spectrom.* **2025**, *40*, 70–97.

(37) Leonori, D.; Sturgeon, R. E. A unified approach to mechanistic aspects of photochemical vapor generation. *J. Anal. At. Spectrom.* **2019**, *34*, 636–654.

(38) Sturgeon, R. E.; Grinberg, P. Some speculations on the mechanisms of photochemical vapor generation. *J. Anal. At. Spectrom.* **2012,** *27*, 222–231.

(39) Vanýsek, P. *CRC Handbook of Chemistry and Physics*. CRC Press*,* 2012.

(40) Sasaki, Y.; Kaneko, M.; Ban, Y.; Kinoshita, R.; Matsumiya, M.; Shinoku, K.; Shiroishi, H. Extraction of Rh(III) from hydrochloric acid by protonated NTAamide(C6) and analogous compounds and understanding of extraction equilibria by using UV spectroscopy and DFT calculations. *Anal. Sci.* **2023**, *39*, 1575–1583.

(41) Shlenskaya, V. I.; Efremenko, O. A.; Oleinikova, S. V.; Alimarin, I. P. Chloride complexes of rhodium (III) in aqueous solutions. *Bull. Acad. Sci. USSR, Div. Chem. Sci.* **1969**, *18*, 1525–1527.

(42) Wolsey, W. C.; Reynolds, C. A.; Kleinberg, J. Complexes in Rhodium(III)-Chloride System in Acid Solution. *Inorg. Chem.* **1963**, *2*, 463–468.

(43) Samuels, A. C.; Boele, C. A.; Bennett, K. T.; Clark, S. B.; Wall, N. A.; Clark, A. E. Integrated Computational and Experimental Protocol for Understanding Rh(III) Speciation in Hydrochloric and Nitric Acid Solutions. *Inorg. Chem.* **2014**, *53*, 12315–12322.

(44) Vasilchenko, D.; Vorob'eva, S.; Tkachev, S.; Baidina, I.; Belyaev, A.; Korenev, S.; Solovyov, L.; Vasiliev, A. Rhodium(III) Speciation in Concentrated Nitric Acid Solutions. *Eur. J. Inorg. Chem.* **2016**, *2016*, 3822–3828.

(45) Watanabe, S.; Sato, T.; Harigai, M.; Inaba, Y.; Takeshita, K.; Onoe, J. Chemical forms of rhodium ion in pure water and nitric acid solution studied using ultraviolet-visible spectroscopy and first-principles calculations. *IOP Conf. Ser. Mater. Sci. Eng.* **2020**, *835*, No. 012001.

(46) Dreher, T. M.; Demopoulos, G. P. The conversion of Rh(III) chlorocomplexes to bromocomplexes and their solvent extraction behaviour. *Solvent Extr. Ion Exch.* **1999**, *17*, 1231–1253.

(47) Castillo-Blum, S. E.; Sykes, A. G.; Gamsjäger, H. Substitution inertness of [Ir(H2O)6]3+. *Polyhedron* **1987**, *6*, 101–103.

(48) Zoschke, K.; Börnick, H.; Worch, E. Vacuum-UV radiation at 185 nm in water treatment – A review. *Water Res.* **2014**, *52*, 131–145.

(49) Lopes, G. S.; Sturgeon, R. E.; Grinberg, P.; Pagliano, E. Evaluation of approaches to the abatement of nitrate interference with photochemical vapor generation. *J. Anal. At. Spectrom.* **2017**, *32*, 2378–2390.

(50) Guo, X.; Sturgeon, R. E.; Mester, Z.; Gardener, G. K. Photochemical alkylation of inorganic selenium in the presence of low molecular weight organic acids. *Environ. Sci. Technol.* **2003**, *37*, 5645–5650.

(51) Guo, X.; Sturgeon, R. E.; Mester, Z.; Gardner, G. J. UV light-mediated alkylation of inorganic selenium. *Appl. Organomet. Chem.* **2003**, *17*, 575–579.

(52) Guo, X.; Sturgeon, R. E.; Mester, Z.; Gardner, G. J. UV vapor generation for determination of selenium by heated quartz tube atomic absorption spectrometry. *Anal. Chem.* **2003**, *75*, 2092–2099.

(53) Ershov, B. G.; Panich, N. M. Chemical and spectral behavior of nitric acid in aqueous sulfuric acid solutions: Absorption spectrum and molar absorption coefficient of nitronium ion. *Spectrochim. Acta, Part A* **2018**, *188*, 179–182.

(54) Duca, C.; Imoberdorf, G.; Mohseni, M. Effects of inorganics on the degradation of micropollutants with vacuum UV (VUV) advanced oxidation. *J. Environ. Sci. Health A* **2017**, *52*, 524–532.

(55) Serrano Mora, A.; Mohseni, M. Temperature dependence of the absorbance of 185 nm photons by water and commonly occurring solutes and its influence on the VUV advanced oxidation process. *Environ. Sci.: Water Res. Technol.* **2018**, *4*, 1303–1309.

(56) Weeks, J. L.; Meaburn, G. M. A. C.; Gordon, S. Absorption Coefficients of Liquid Water and Aqueous Solutions in the Far Ultraviolet. *Radiat. Res.* **1963**, *19*, 559–567.

(57) Qin, D.; Gao, F.; Zhang, Z.; Zhao, L.; Liu, J.; Ye, J.; Li, J.; Zheng, F. Ultraviolet vapor generation atomic fluorescence spectrometric determination of mercury in natural water with enrichment by on-line solid phase extraction. *Spectrochim. Acta, Part B* **2013**, *88*, 10–14.

(58) Campanella, B.; Menciassi, A.; Onor, M.; Ferrari, C.; Bramanti, E.; D'Ulivo, A. Studies on photochemical vapor generation of selenium with germicidal low power ultraviolet mercury lamp. *Spectrochim. Acta, Part B* **2016**, *126*, 11–16.

(59) Zwinkels, J.; Lee, S. *UV transmittance spectrum of Teflon tubing, Unpublished data*. NRCC, October 2017.
